# Reduction of Nitrate Content in Baby-Leaf Lettuce and *Cichorium endivia* Through the Soilless Cultivation System, Electrical Conductivity and Management of Nutrient Solution

**DOI:** 10.3389/fpls.2021.645671

**Published:** 2021-04-29

**Authors:** Giulia Conversa, Anna Bonasia, Corrado Lazzizera, Paolo La Rotonda, Antonio Elia

**Affiliations:** Department of Agriculture, Food, Natural Resources and Engineering, University of Foggia, Foggia, Italy

**Keywords:** ebb and flow, floating, salinity, final nutrient solution withdrawal, endive, appearance, nitrates, antioxidants

## Abstract

Soilless cultivation systems are efficient tools to control nitrates by managing nutrient solution (NS) salinity and nitrogen availability, however, these nitrate-lowering strategies require appropriate calibration based on species/genotype-specific responses interacting with climate and growing conditions. Three experiments were carried out on lettuce and *Cichorium endivia* grown in ebb-and-flow (EF) and floating (FL) systems at two levels of NS salinity (EC = 2.5 and 3.5 dS m^−1^) (EC2.5, EC3.5, respectively) under autumn and early-spring (lettuce) and winter and late-spring conditions (*C. endivia*). Nitrogen deprivation (NS withdrawal a few days before the harvest) was tested at EC2.5, in the autumn and winter cycles. The EF-system caused an increase in salinity in the substrate where roots mainly develop so it mimicked the effect of the EC3.5 treatment. In the winter-grown lettuce, the EF-system or EC3.5 treatment was effective in reducing the nitrate level without effects on yield, with the EF baby-leaf showing an improved quality (color, dry matter, chlorophylls, carotenoid, vitamin C, phenol). In both seasons, the EF/EC3.5 treatment resulted in a decline in productivity, despite a further reduction in nitrate content and a rise in product quality occurring. This response was strictly linked to the increasing salt-stress loaded by the EC3.5/EF as highlighted by the concurrent Cl^−^ accumulation. In early-spring, the FL/EC3.5 combination may represent a trade-off between yield, nitrate content and product quality. In contrast, in winter-grown endive/escarole the EC3.5, EF and EC3.5/EF reduced the nitrate level with no effect on yield, product quality or Cl^−^ uptake, thus proving them to be more salt-tolerant than lettuce. High temperatures during the late-spring cycle promoted nitrate and Cl^−^ uptake, overcoming the nitrate-controlling effect of salinity charged by the EF system or EC3.5. The nitrate level decreased after 3 day-long (lettuce) or 6 day-long (*C. endivia*) NS withdrawal. In *C. endivia* and EF-grown lettuce, it provoked a decrease in yield, but a concurrent improvement in baby-leaf appearance and nutritional quality. More insights are needed to fine-tune the duration of the NS removal taking into account the soilless system used and species-specific characteristics.

## Introduction

The baby-leaf category is important among the leafy vegetables. It is harvested at an early-vegetative phase (8–12 cm in length), hence the name, and includes many species, with *Lactuca sativa* L. and *Cichorium endivia* L. varieties among the most popular (Nicola and Fontana, 2014). They are mainly used as minimally processed vegetable products (Conesa et al., 2015) and consumed in increasing amounts as they provide an important source of health-promoting compounds such as carotenoids, vitamin C, and polyphenols (DuPont et al., 2000; El-Nakhel et al., 2019).

Nevertheless, lettuce, endive (*Cichorium endivia* L. var *crispum* Hegi) and escarole (*Cichorium endivia* L. var *latifolium* Hegi) are included among the greatest accumulators of undesirable nitrates in leafy vegetables and, as such, they are a potential threat to consumer health (Santamaria, 2006; Kmecl et al., 2017). In particular, for lettuce a wide variability in nitrate accumulation has been proved according to morphotype, with romaine lettuce showing a lower content both in winter and summer cycles compared with butterhead, curled leaf and crisphead lettuces (Burns et al., 2011a,b). Romaine lettuce has also been reported to be less prone to accumulate nitrate than oak-leaf lettuce in fall-winter cycles (Di Gioia et al., 2017).

Although it has not been scientifically demonstrated, the European Union as a precautionary measure has set restriction limits to some commercialized leafy vegetable such as lettuce (European Community Regulation 1258/2011) while some European countries (Austria, Belgium, Netherlands, Switzerland) have adopted national limits for the internal market for endive and escarole (Santamaria, 2006).

Soilless cultivation systems (SCS) are rather common for baby-leaf production and in particular, hydroponics provides efficient tools to manage nitrates through complete control of nitrogen nutrition via the nutrient solution (NS). The nitrate level in leafy vegetables is reported to be efficiently decreased by reducing N-nitrate availability in the NS (Santamaria et al., 1998; Bonasia et al., 2008) or by simply replacing NS with freshwater a few days before harvest. Previous experiences on NS withdrawal have been carried out on endive (Elia et al., 1999), rocket salad (Santamaria et al., 2001), lambs' lettuce (Gonnella et al., 2004) and cardoon (Borgognone et al., 2016). They report a significant decrease in nitrates with no detrimental effects on yield, but they highlight the very scarce information about the effects on product quality.

Besides nitrogen, other nutrients influencing nitrate accumulation can also be managed in hydroponic crops. A nitrate content reduction has been reported for lettuce (Serio et al., 2001; Scuderi et al., 2011) and rocket (Barbieri et al., 2011; Bonasia et al., 2017) grown under saline conditions as the ${\text{NO}}_{3}^{-}$ uptake is inhibited by chloride (Cl^−^) (Rouphael and Kyriacou, 2018). The salinity stress also improved the visual (color, firmness) (Scuderi et al., 2011; Bonasia et al., 2017) and nutritional quality of these baby-leaves (Bonasia et al., 2017), however when NS salinity was higher than the tolerance threshold of crops it negatively affected the yield.

It is well-known that plants may differently accumulate nitrates in relation to climate (Santamaria, 2006), showing lower levels with a higher sunlight availability and temperature regime, so the control of nitrates becomes more challenging in the autumn and winter cycles during the growing season in lettuce (Fallovo et al., 2009; Bonasia et al., 2013; Sublett et al., 2018) and other leafy vegetables (Conte et al., 2008; Conversa et al., 2016; Bonasia et al., 2017).

The hydroponic floating system (FL) is most widely used for the production of high-quality, minimally processed vegetables as it is an easy, resource-saving and profitable growing technique (Tomasi et al., 2015; Sambo et al., 2019). However, some concerns arise about this system as it is static (with no recirculation of NS) and a lack of oxygen frequently occurs in the NS, especially at high temperatures (Conesa et al., 2015) so farmers are forced to provide continuous oxygen enrichment of the NS. The oxygen deficiency may reduce crop yield, leaf appearance (color) and its nitrate level, whereas it could enhance product antioxidative proprieties (Conesa et al., 2015). To cope with this potential limit, the ebb and flow system (EF) can be used as an alternative for growing baby-leaf vegetables as it allows better root oxygenation deriving from the periodical NS supply to the root through sub-irrigation. The information available on the effect of EF is only limited to vegetables (Nicola et al., 2003; Rouphael and Colla, 2005; Hamilton and Fonseca, 2010) other than lettuce, endivia and escarole and the EF system has only been tested for wild rocket for imposing saline stress (Bonasia et al., 2017). Moreover, no published data are available on the NS replacement with water for baby-leaf production in floating in comparison with the ebb and flow system.

The above-mentioned nitrate-lowering strategies might exhibit changing efficiency in relation to species/genotype-specific responses, the climate and the growing conditions imposed by the SCS and NS management. In order to identify the best soilless approach to control nitrate in two high-nitrate accumulating species, this work aims to assess the effect of: (a) ebb and flow and floating soilless cultivation systems; (b) nutrient solution salinity; (c) the withdrawal of the nutrient solution a few days before the harvest; and d) the growing cycle on the nitrate content and nutritional traits, the growth and yield, as well as the bio-morphological traits of genotypes of baby-leaf romaine lettuce and *C. endivia*.

## Materials and Methods

### Crop and Trial Set-Up

Three experiments were carried out in the 2013–2014 period using the species *Lactuca sativa* var. *longifolia* L. and *Cichorium endivia* L. grown in soilless cultivation systems in an unheated greenhouse. The greenhouse was covered with wavy methyl polymethacrylate (Ondex, Renolit Milano S.r.l, Peschiera borromeo, MI, Italy), and was located in Foggia (Puglia region, Southern Italy, latitude 41° 46' N, longitude 15° 55' E, 74 m a.s.l.). The treatment details of the different trials carried out are summarized in Table 1. Plants were raised from seeds in polystyrene trays (336 cells) filled with perlite (Agrilit 3, Perlite Italiana S.r.l., Corsico, MI, Italy) at 1,896 plants per m^2^. After irrigation with tap water (pH 6.8 ± 0.2 and EC 0.7 ± 0.2 dS m^−1^), all trays were placed in a growth chamber (Piardi, Brescia, Italia) until emergence. The growth chamber was set at 20°C day/night and 70–80% relative humidity in the dark for 2 days following by 12 h of photoperiod, and 190 ± 10 μmol m^−2^ s^−1^ photosynthetically active photons. Irradiance illumination was supplied by Lumilux fluorescent lamps (Osram L 36 W/840-1).

**Table 1:** Treatment details for the different experiments.

**Species**	**Botanical variety**	**Common name**	**Cultivar name**	**Soilless cultivation system**	**Crop cycle**	**EC level (dS m^**−1**^)**	**Nutrient solution management^β^**	**Experiment**
*Lactuca sativa*	*longifolia*	Romaine lettuce	Lastra	Ebb and flow	Autumn	2.5	noWD WD	1, 2 2
						3.5	noWD	1, 3
			Green forest		Early spring	3.5	noWD	3
*Cichorium endivia*	*crispum*	Endive	Atleta	Floating	Winter	2.5	noWD WD	1, 2 2
						3.5	noWD	1, 3
	*latifolium*	Escarole	Bionda a cuore pieno		Late-spring	3.5	noWD	3

β *Nutrient solution management; WD, withdrawal of the nutrient solution and its replacement with freshwater 3 (lettuce) or 6 (C. endivia) days before harvest; noWD, permanence of the same nutrient solution up to harvest*.

At the cotyledon stage (6–8 days after sowing), plantlets were transferred in the greenhouse to be grown in floating (FL) and ebb-and-flow (EF) soilless cultivation systems (SCS).

In both SCSs the set-up consisted of aluminum benches (256 cm long, 96 cm wide, with a 5 cm high border). Each bench was connected through a pump to a 100 L water tank positioned below, which was used for NS replenishment or movement.

In the FL system, the NS was always maintained on the bench (50 L, ~2 cm of water height), except for a daily movement of NS between the bench and the tank below for oxygen enrichment (emptying and refilling of the bench).

In the EF system, the trays were laid on the benches and were periodically sub-irrigated with a 3-min flow of NS through the benches at the base of trays, three times a day in the autumn and winter cycles and five times a day in the early- and late-spring cycles every 100 min starting at 8:00 a.m.

With both SCSs a total 50 L of NS was maintained throughout the cycle by replenishment with new NS every 2 days.

The concentrations of the nutrients in the basic NS were 140 (10 mM), 50 (1.6 mM), 200 (5.1 mM) 100 (2.5 mM), 38.4 (1.6 mM) and 136 (4.2 mM) mg L^−1^ of N, P, K, Ca, Mg and S, respectively, with a NO_3_:NH_4_ ratio of 4:1. Microelements were used at the concentrations reported for Hoagland solution.

**Figure 1:**
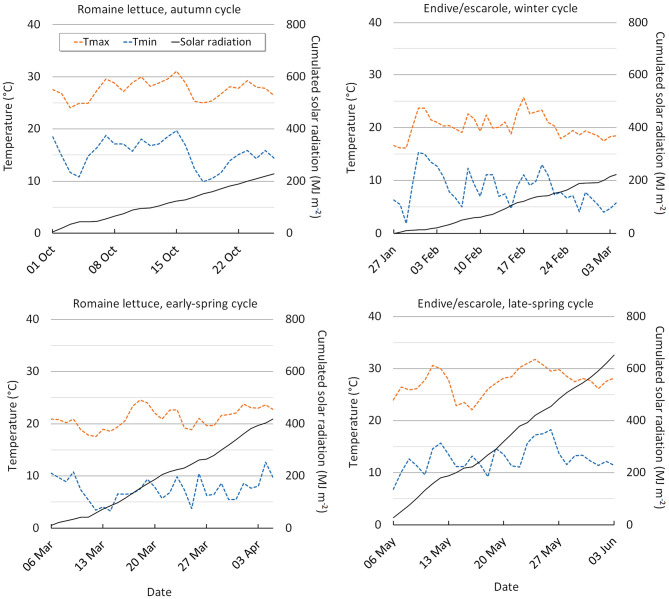
Internal greenhouse minimum and maximum air temperature, and cumulated solar radiation during the autumn, early-spring (lettuce), winter and late-spring periods (*C. endivia*).

The EC, the dissolved O_2_ and the pH of the NS were checked every 2 days. The pH was maintained between 5.5 and 6.5, through the addition of 1M HCl. The EC and the pH of the NS were measured using a hand-held conductivity and pH-meter (Hanna Instruments Italia s.r.l., Villafranca, PD, Italy) and the dissolved O_2_ (mg L^−1^) was measured with a hand-held oximeter (Crison Strumenti S.p.a, Oxi45+, Carpi, MO, Italy). The mean values of the nutrient solution EC and dissolved O_2_ measured during the crop cycles are reported in Supplementary Table 1. The climatic conditions recorded in the trial periods are reported in Figure 1.

### Experiment 1

Two cultivars of romaine lettuce (Lastra and Green Forest -Royal Seed, MO, Italy) were sown on 1^th^ October 2013 (autumn cycle, A) and two botanical varieties of *C. endivia* (var. *latifolium* Hegi, cultivar Bionda a cuore pieno -Royal Seed) and var. *crispum* Hegi, cultivar Atleta - Enza Zaden Tarquinia, VT, Italy) were sown on 27^th^ January 2014 (winter cycle, W). The harvests at the baby-leaf stage took place on 26^th^ October, 2013 (25 days after sowing) and on 4^th^ March 2014 (36 days after sowing) in the autumn and winter cycles, respectively.

For both species, the treatments were (i) two soilless cultivation systems: floating (FL) and ebb and flow (EF), (ii) two levels of electrical conductivity (EC) of the nutrient solution (NS): 2.5 dS m^−1^ (EC2.5) and 3.5 dS m^−1^ (EC3.5), (iii) two genotypes.

A split-split plot experimental design was adopted with three replications and with the soilless cultivation system as main plots, the level of EC (one bench with 16 trays) as sub-plots, the genotype as sub-sub-plots (8 trays per genotype on each bench) (the experimental unit).

The different salinity levels were obtained by adding 1.7 and 12 mmol L^−1^ of NaCl to the basal NS (EC 2.41 dS m^−1^) for the EC2.5 and EC3.5 treatment, respectively.

#### Sampling and Measurements

Plants were harvested at the optimal stage for fresh consumption as baby leaves (~12 cm long) by cutting leaves at about 1 cm above the collar. The raw material was directly transported to the laboratory at the Department of Agriculture, Food, Natural resources and Engineering (DAFNE) (~1 km away) and immediately processed within 1 h after harvest.

Productive, biophysical, physiological (fresh and dry weight, leaf number, leaf height, leaf area, dry matter concentration, specific leaf area, main color indices, chlorophyll content, relative water content, electrolytic leakage) and nutritional parameters (nitrate, chloride, total phenol and carotenoid concentrations) were determined for all genotypes. All samples were analyzed in three replicates except for color (20 replicates).

#### Yield, Morphological and Biophysiological Measurements

Fresh weight (FW) and dry weight (DW) were calculated by considering the whole experimental unit. After the harvest, leaves from each plot were well-mixed to obtain a homogeneous sample for measurements. The dry matter concentration (DM) was calculated as dry weight (DW)/fresh weight (FW)^*^100. In order to determine the DW, fresh plant material was dried in a thermo-ventilated oven at 70°C until it reached a constant mass. Leaf area was measured on a sample of 30 plants for each treatment using LI-COR 3100 (LICOR, Lincoln, NE, USA). The Specific Leaf Area (SLA) was expressed as DW/leaf area (mg cm^−2^).

The leaf color indices were measured on fresh material using a portable tristimulus color-meter (Minolta Chroma Meter CR-200; Minolta Camera Co. Ltd., Osaka, Japan), using the CIE-L^*^a^*^b^*^ scale 1976. The chroma meter was calibrated using a standard white color, and the color was expressed in the tristimulus L^*^ (lightness), a^*^ (green to red), and b^*^ (yellow to blue), from which hue angle (h°) and Chroma were calculated.

The relative water content (RWC) was determined on fresh leaf blade discs. The sample was first weighed to determine the fresh weight (FW) and then it was hydrated to full turgidity for 24 h, under normal room light and temperature conditions, in de-ionized water in a closed Petri dish. Then the sample was taken out of the water and well-dried off with filter paper and immediately weighed to obtain the fully turgid weight (TW). The sample was then oven-dried at 70°C and weighed to determine the dry weight (DW). The RWC was estimated from the equation: RWC = (FW– DW)/(TW – DW)^*^100.

The electrolyte leakage (EL) was determined according to the method of Yan et al. (1996). A portion of fresh leaf material (3 g) was weighed in a glass beaker containing twice-distilled water. The electrical conductivity of the solution (EC1) was measured using a hand-held conductivity-meter (Hanna Instruments Italia s.r.l., Villafranca, PD, Italy). After boiling the sample for 2 min and cooling it to room temperature, the electrical conductivity of the solution was re-measured (EC2). The percentage of electrolyte leakage was calculated as EL (%) = (EC1/EC2)^*^100.

The total chlorophyll (CHLtot) (CHLa + CHLb) was extracted from previously frozen samples by homogenizing in 80% acetone, spectrophotometrically measured and estimated using the equation of Dere et al. (1998) and expressed on a fresh weight basis.

#### Nutritional Measurements

The concentrations of inorganic anions, total phenols, vitamin C and carotenoids were determined from frozen plant material successively lyophilized and then ground into fine particles.

Anions were extracted from 0.5 g of sample with 50 mL of 3.5 mM sodium carbonate and 1 mM sodium bicarbonate solution in a shaking water bath at room temperature for 30 min. The mixture was filtered through Whatman n. 2 paper. The filtrates were filtered again through 0.22 μM Millipore filter, before injection into the ion chromatography system (Dionex ICS 3000, Dionex-ThermoFisher Scientific, Waltham, MA, USA). The system was equipped with: an isocratic pump, a model AS-DV autosampler, a self-generating ASR anion suppressor (4 mm), A Dionex Ion-Pac AS23 (Dionex ICS 3000, Dionex-ThermoFisher Scientific, Waltham, MA, USA) analytical column (4 × 250 mm) and a guard column (4 × 50 mm) maintained at 35°C. The eluent corresponded to the extraction solution used at a flow rate of 1 mL min^−1^. The anions were identified by comparison of the retention times with those of standards. Peak areas were analyzed using Dionex-ThermoFisher Scientific, Waltham, MA, USA Chromeleon software (version 6.80, Thermo Scientific).

Total phenols were extracted from 30 mg of the sample with 1 mL of water/methanol (20:80, v/v) at room temperature in a shaking water bath (100 rpm, 25°C) for 15 min; then the mixture was centrifuged in a refrigerated centrifuge (ThermoFisher Scientific, Waltham, MA, USA) (14,000 rpm for 15 min at 4°C) and the supernatant was collected. The extraction was repeated once and two supernatants were combined. The extracts were stored at −20°C and measured within 24 h. Total phenol concentration was determined on methanolic extracts as reported in Bonasia et al. (2013). Briefly, 100 μL of the extracts was diluted with 3 mL of distilled water, mixed with 0.5 mL of Folin–Ciocalteu reagent and kept at room temperature for 5 min; then 1.0 mL of 20% Na_2_CO_3_ was added to the mixture. After 45 min at 30°C, absorbance was read at 750 nm (Shimatzu UV-1800, Shimadzu Scientific Instruments, North America, USA). The results are expressed as gallic acid equivalents (GAE) (mg 100 g^−1^ FW) using a calibration curve.

Vitamin C was extracted according to the modified method of Koh et al. (2009). In order to determine the total concentration of vitamin C (ascorbic acid + dehydro-ascorbic acid), dehydro-ascorbic acid was reduced to ascorbic acid (AA) with dithiothreitol (DTT). Reduced samples were injected into the chromatographic system. The ion chromatography instrument equipment (ICS 3000 System, Dionex) included: a 10 μL injection loop, C18 - 5 μm reverse-phase ion-exchange columns (Acclaim 120, Dionex-ThermoFisher Scientific, Waltham, MA, USA) combined with a UV-visible detector (RLSC Diode Array Detector, Dionex). AA was identified and quantified by retention time and spectra. The mobile phase was 0.05 M monobasic potassium phosphate buffer (KH_2_PO_4_) adjusted to pH 4.5 for the first 6 min, gradually followed by buffer and ethanol in a 60:40 ratio from the 6th to the 10th min; 1 min to return to 100% buffer, final 5 min at 100% buffer.

The flow rate was fixed at 1 mL min^−1^; the temperature of the column was set at 30°C. The detection wavelength was 254 nm and the UV spectra were in the 190–350 nm range. The method was calibrated with a curve of standard AA solution.

Carotenoids were extracted from 0.1 g of the sample (plus 0.05 g of MgCO_3_ to neutralize cytosolic acids and 0.01 g of celite for better tissue disruption) with 10 mL of ethanol:hexane (4:3 by volume). The pyrogallol solution (5%) (1 mL) was added as an antioxidant. The mixture was placed in a mechanical shaker for 15 min, then centrifuged at 6,700 rpm for 10 min and the supernatant was collected. The residue was re-extracted; the two extracts were combined and decanted into a 50-mL tube. The supernatant hexane phase was transferred into another tube, and the lower aqueous phase was discarded. To overcome the problem of carotenoid overestimation by the presence of chlorophyll, a saponification step was included during extraction. In brief, an equal volume of 10% methanolic KOH was added to the recuperated hexane phase, the mixture was shaken vigorously for 1 min and placed in ice for 15 min. After centrifuging at 6,700 rpm for 10 min, the supernatant (hexane phase) was collected and washed 2 times with 15 of NaCl 10% solution and two times with 15 ml water. The aqueous phase was discarded. All samples were stored at −25°C until analysis. The total carotenoid present in the extract was measured at 450 nm by UV-visible spectrophotometer (Shimadzu UV-1800) and estimated according to “Method of Mean,” reported by Biehler et al. (2009).

#### Chemicals and Standards

Acetone, sodium carbonate, sodium bicarbonate, magnesium carbonate, potassium hydroxide, methanol, gallic acid reagent and ultrapure water were purchased from Carlo Erba (Rodano, MI, Italy). Dithiothreitol, ethanol, hexane, Folin–Ciocalteu reagent, celite, pyrogallol and sodium chloride were purchased from Sigma-Aldrich (Milan, Italy). Ascorbic acid and monobasic potassium were purchased from Mallinckrodt Baker B.V. (Deventer, Netherlands).

### Experiment 2

This experiment was performed simultaneously with experiment 1. Both genotypes of romaine lettuce and *C. endivia* were grown in FL and EF systems with a nutrient solution at EC 2.5 dS m^−1^ to be submitted at the substitution of NS with freshwater (NS withdrawal- WD) a few days before the harvest. The plants grown in the EF and FL system at 2.5 EC level in experiment 1 were used as controls (noWD). The NS for the EC2.5 treatment was obtained as reported in experiment 1.

The experimental design was a split-split plot with three replications with the SCSs as main plots, the NS management (WD and noWD) (one bench with 16 trays) as subplots, and genotype as sub-sub-plots (8 trays per cultivar or botanical variety on each bench) (the experimental unit). The benches of NS-WD were fed with NS until three (for lettuce) or six (for *C. endivia*) days before harvest when it was replaced with fresh water (pH 6.3 ± 0.2 and EC 0.5 ± 0.4 dS m^−1^).

The sowing and harvest times, the cultivation system set-up and management are as described in experiment 1.

For lettuce, the fresh weight (FW), dry weight (DW), dry mass concentration (DM), and anion concentrations were determined, whereas for *C. endivia* all the measurements described in experiment 1 were performed.

### Experiment 3

To evaluate the effect of the growing season, experiment 1 was repeated (the same genotypes for both lettuce and *C. endivia*, grown in FL and EF systems) but only at 3.5 dS m^−1^ EC (EC3.5) during an early-spring (ES) (lettuce) or late-spring (LS) (*C. endivia*) cycle. The NS saline treatment was obtained as reported in experiment 1.

The experimental design was a split-plot with three replications with the SCS as main plots and the two genotypes as sub-plot. The autumn and the winter trials were part of experiment 1 so the details were as described above.

In the early-spring cycle, lettuce was sown on 6th March 2014 and harvested on 5^th^ April (30 days after sowing) whereas in the late-spring trial, *C. endivia* was sown on 6th May 2014 and was harvested on 3^rd^ June 2014 (28 days after sowing). All the measurements described in experiment 1 were performed.

### Statistical Analysis

All data were statistically analyzed by ANOVA carried out using GLM (General Linear Model) procedure - SAS software. In experiment 3, a combined analysis of variance was performed using the season as a fixed variable. The least significant difference (LSD) test (*P* = 0.05) was used to establish differences between means.

## Results and Discussion

### Effects of Soilless Cultivation System, Nutrient Solution Salinity and Genotype (Experiment 1)

#### Growth, Yield, Leaf Bio-Physiological Traits

##### Lactuca sativa var. longifolia

Both with the ebb and flow system (EF) and with the highest salinity of nutrient solution (NS) (EC3.5) a reduced plant dry weight (DW) (−8%, on average) occurred, along with a rise in dry matter concentration (DM) in comparison with FL and EC2.5 plants. Plants grown in EF or at EC3.5 also exhibited a very similar reduction in leaf area, leaf number and, only with EC3.5, in height (Table 2).

**Table 2:** Effect of soilless cultivation system (SCS), salinity level of nutrient solution (EC), and genotype (G) on yield and bio-morphological traits of romaine lettuce (autumn cycle) and *C. endivia* (winter cycle) leaves, with the standard error of the mean in brackets.

**Treatments**	**Fresh weight (kg m^**−2**^)**	**Dry weight (g m^**−2**^)**	**Dry mass (g kg^**−1**^ FW)**	**Area (cm^**2**^)**	**Height (cm)**	**Number (*n*.)**	**Specific area (mg cm^**−2**^)**
**Romaine lettuce**							
SCS^†^							
EF	1.6 (0.1)^b^	84.2 (4.2)^b^	53.8 (0.2)^a^	35.2 (0.8)^b^	14.8 (0.1)^b^	4.1 (0.02)^b^	1.9 (0.10)^a^
FL	2.1 (0.1)^a^	90.4 (4.1)^a^	43.4 (0.1)^b^	38.0 (1.0)^a^	15.5 (0.1)^a^	4.5 (0.03)^a^	1.5 (0.03)^b^
Salinity level (EC)							
2.5 dS·m^−1^	2.0 (0.1)^a^	91.6 (3.8)^a^	44.7 (0.1)^b^	39.0 (0.7)^a^	16.0 (0.1)^a^	4.4 (0.03)^a^	1.6 (0.04)^b^
3.5 dS·m^−1^	1.6 (0.1)^b^	83.4 (4.5)^b^	52.5 (0.2)^a^	34.1 (0.9)^b^	14.3 (0.1)^b^	4.2 (0.03)^b^	1.7 (0.10)^a^
Genotype (G)							
Lastra	1.4 (0.1)^b^	68.4 (1.6)^b^	48.8 (0.1)^a^	35.0 (1.0)^b^	13.9 (0.1)^b^	4.4 (0.03)^a^	1.5 (0.03)^b^
Green Forest	2.3 (0.1)^a^	104.6 (2.2)^a^	48.3 (0.2)^a^	38.1 (0.8)^a^	16.3 (0.1)^a^	4.2 (0.03)^b^	1.7 (0.10)^a^
Significance^††^							
SCS	^***^	^**^	^***^	^**^	^***^	^***^	^***^
EC	^***^	^*^	^**^	^***^	^***^	^**^	^*^
G	^***^	^***^	ns	^**^	^***^	^***^	^***^
SCSxEC	^***^	ns	^***^	ns	ns	ns	^*^
***Cichorium endivia***							
SCS^†^							
EF	2.0 (0.1)^a^	112.8 (5.4)^a^	55.4 (0.1)^a^	32.8 (1.6)^b^	13.3 (0.2)^a^	3.8 (0.1)^b^	2.0 (0.1)^a^
FL	2.0 (0.1)^a^	111.6 (5.3)^a^	55.3 (0.1)^a^	39.3 (1.8)^a^	13.4 (0.1)^a^	4.1 (0.1)^a^	2.0 (0.1)^a^
Salinity level (EC)							
2.5 dS·m^−1^	2.1 (0.1)^a^	118.0 (5.2)^a^	56.1 (0.1)^a^	37.6 (2.4)^a^	13.7 (0.1)^a^	3.9 (0.1)^a^	2.0 (0.1)^a^
3.5 dS·m^−1^	2.0 (0.1)^a^	106.5 (4.9)^a^	54.6 (0.1)^a^	34.5 (1.3)^a^	13.0 (0.2)^b^	4.0 (0.1)^a^	2.0 (0.1)^a^
Genotypes (G)							
Endive	1.9 (0.1)^a^	109.0 (4.5)^a^	56.7 (0.1)^a^	39.1 (1.6)^a^	13.8 (0.1)^a^	4.4 (0.1)^a^	1.9 (0.04)^b^
Escarole	2.1 (0.1)^a^	115.5 (5.9)^a^	54.0 (0.1)^b^	33.0 (1.9)^b^	12.9 (0.1)^b^	3.6 (0.1)^b^	2.1 (0.1)^a^
Significance^††^							
SCS	ns	ns	ns	^**^	ns	^**^	ns
EC	ns	ns	ns	ns	^***^	ns	ns
G	ns	ns	^*^	^**^	^***^	^***^	^*^

† *SCS, Soilless Cultivation System; EF, Ebb and flow system; FL, Floating system*.

†† *ns*,

^*^, ^**^, and ^***^, not significant or significant at P ≤ 0.05, P ≤ 0.01, or P ≤ 0.001, respectively.

*The SCSxG, ECxG and SCSxECxG interactions in lettuce, and SCSxEC, SCSxG, ECxG and SCSxECxG interactions in C. endivia were never significant*.

a,b *Means in columns (and by effect) not sharing the same letters are significantly different according to the LSD test (α = 0.05)*.

The averaged dissolved oxygen concentration, measured during the crop cycle, was quite high, with small differences between EF and FL (Supplementary Table 1), so in both cases no hypoxic stress affecting plant growth (Tesi et al., 2003) is conceivable. Moreover, the average electrical conductivity of NS during the crop cycle showed negligible differences between the EF and FL systems, with even higher values in this latter (Supplementary Table 1). These results suggest that in the EF system the partial drying between the intermittent wettings (3-min wetting flux at the base of the trays every 100 min) exacerbated salt accumulation in the substrate where roots mainly developed, so mirroring the effect of the EC3.5 treatment on shoot growth. On the contrary, the root apparatus in the FL system was always immersed in the NS with a more stable level of electrical conductivity maintained during the cycle.

The above-described plant responses are expected since a general decrease in fresh and/or dry weight is reported in all plant tissues subjected to salt stress, especially in the aerial part with a reduction in the number and area of leaves, due to a decrease in water potential (osmotic stress) in the growing medium (soil/substrate or NS) (Xu and Mou, 2015; Acosta-Motos et al., 2017). A substantial reduction in dry weight has been reported in romaine lettuce when irrigated for 15 days with very high saline water (>100 mM NaCl) (Kim et al., 2008) or grown in a soilless system with high saline nutrient solution (>100 mM NaCl) (Mahmoudi et al., 2010). When hydroponically grown at 6.3 dS m^−1^ EC of NS (with NaCl and CaCl added in a 2:1 ratio), in cultivars of crisphead, butterhead and romaine lettuce the dry weight decreased to an even greater extent (−19, –50%) (Adhikari et al., 2019) than that observed in our study.

**Figure 2:**
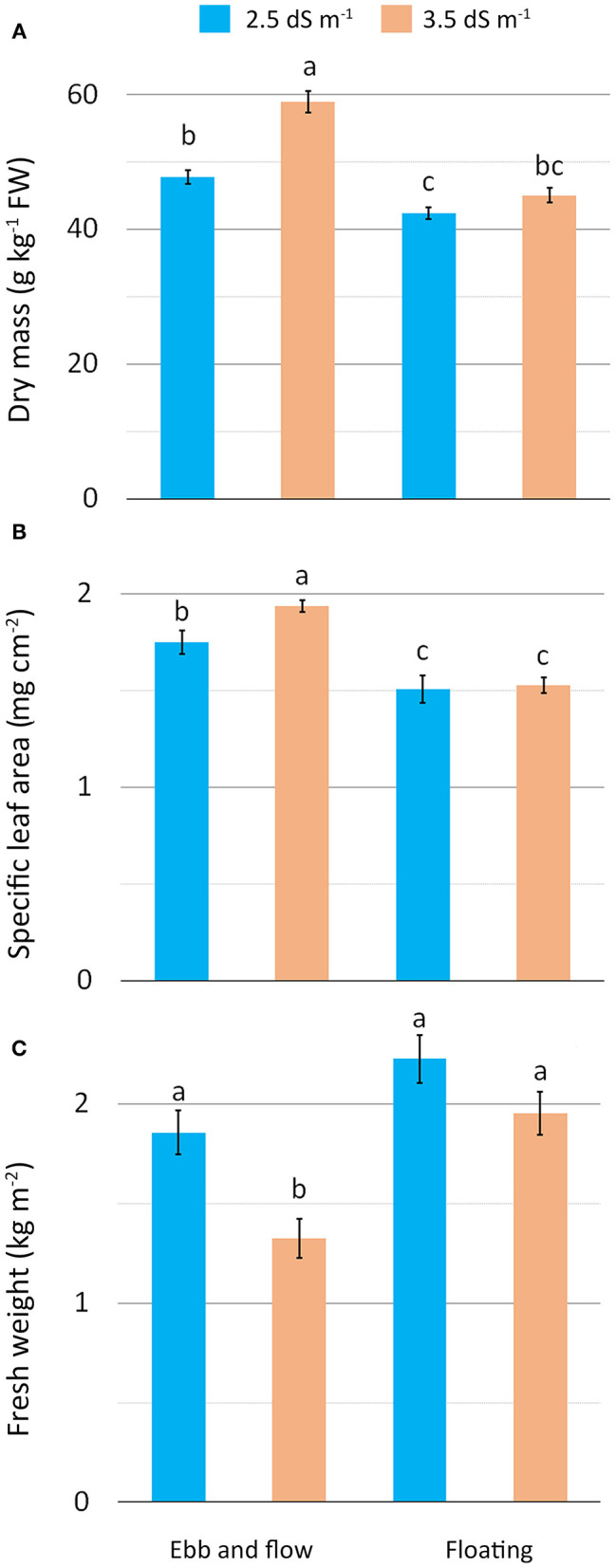
Effect of soilless cultivation system and nutrient solution electrical conductivity on dry mass **(A)**, specific leaf area **(B)** and yield **(C)** of romaine lettuce grown in a greenhouse during the autumn. Vertical bars indicate ±SE of mean (*n* = 9) of the observed values, with different letters significantly different according to the LSD test (α = 0.05).

The detected increase in leaf DM under the higher saline conditions (EF or EC3.5) may be construed as a plant adjustment to osmotic stress involving the cell accumulation of solutes (inorganic ions and/or organic compounds) aimed to reduce cellular osmotic potential (Barbieri et al., 2011; Acosta-Motos et al., 2017). In our study, this response was even more pronounced when EF was combined with EC3.5 treatment (EF/EC3.5), suggesting that the EF growing system additively acted to raise substrate salinity. The EF/EC3.5 leaves had the highest DM (Figure 2A) along with the highest specific area (SLA) (EF/EC3.5) (Figure 2B) (thicker leaves) compared with the other treatments. Salt-stressed plants respond to salinity by increasing SLA (Acosta-Motos et al., 2017) as also confirmed in romaine lettuce grown in a soilless system at 3.8 and 4.8 dS m^−1^ which showed firmer leaves than at 2.8 dS m^−1^ (Scuderi et al., 2011) and in other leafy vegetables such as wild rocket (Bonasia et al., 2017). The salt-stress that occurred with the combination EF/EC3.5 also resulted in a substantial reduction in plant fresh weight (yield) as a consequence of the lower water content of tissues. In contrast, no significant differences in yield were observed among the other treatments (Figure 2C). Similarly, a drop in fresh yield has been reported in butterhead lettuce at the rosette stage grown in soil with irrigation water at 3.6 and 7.2 dS m^−1^ (Di Mola et al., 2017) and in many lettuce cultivars submitted to salt stress (6.3 dS m^−1^) (Adhikari et al., 2019). These results confirm that in general for lettuce, an acceptable yield performance is expected at EC not higher than 3.6 dS m^−1^ (Atzori et al., 2019). However, the general improvement in DM and SLA obtained with moderate saline stress can be considered a positive effect for baby-leaf vegetables as it enhances their suitability to be processed as fresh-cut (Clarkson et al., 2003; Conversa et al., 2014).

**Figure 3:**
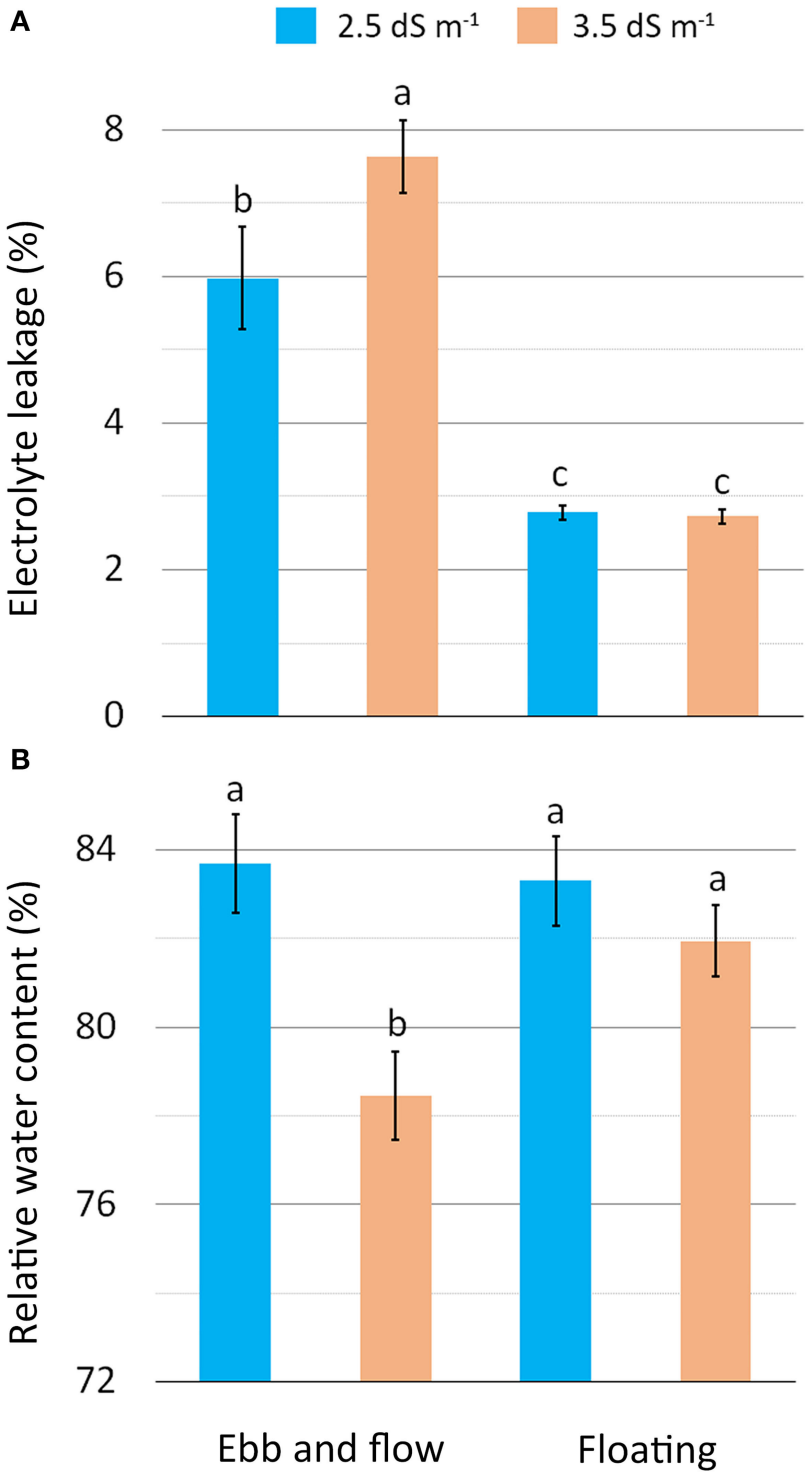
Effect of soilless cultivation system and nutrient solution electrical conductivity on electrolyte leakage **(A)** and relative water content **(B)** of romaine lettuce grown in a greenhouse during the autumn. Vertical bars indicate ±SE of mean (*n* = 9) of the observed values, with different letters significantly different according to the LSD test (α = 0.05).

The physiological status, tested by membrane electrolyte leakage (EL) and leaf relative water content (RWC) (Table 3), reveals more stressed tissues for EF plants with a rise in membrane permeability, particularly in combination with the EC3.5 treatment (Figure 3A). The higher membrane damage observed in EF/EC3.5 plants may be associable with a more pronounced generation of reactive oxygen species (ROS) prompted by the greater level of salinity (Mahmoudi et al., 2010). Additionally, the EF/EC3.5 plants also showed the lowest tissues hydration (RWC) (Figure 3B), confirming the higher salt-stressing conditions which occurred in EF coupled with the highest NS salinity. Other authors have also reported the negative correlation between RWC and the salinity level of the NS for lettuce and sage (Taârit et al., 2012; Garrido et al., 2014).

**Table 3:** Effect of soilless cultivation system (SCS), salinity level (EC) of nutrient solution, and genotype (G) on the bio-physiological traits of romaine lettuce (autumn cycle) and *C. endivia* (winter cycle) leaves, with the standard error of the mean in brackets.

**Treatments**	**Electrolyte leakage (%)**	**Relative water content (%)**	L^*^	**h^**°**^**	**Chroma**	**CHLa**	**CHLb**	**CHLtot (μg mg^**−1**^ FW)**
**Romaine lettuce**								
SCS^†^								
EF	6.8 (0.3)^a^	81.1 (0.9)^a^	52.0 (0.3)^b^	127.2 (0.1)^a^	36.7 (0.4)^b^	0.63 (0.02)^a^	0.20 (0.01)^a^	0.83 (0.02)^a^
FL	2.8 (0.1)^b^	82.7 (0.5)^a^	53.7 (0.3)^a^	126.1 (0.1)^b^	40.1 (0.4)^a^	0.57 (0.02)^b^	0.17 (0.01)^b^	0.75 (0.03)^b^
Salinity level (EC)								
2.5 dS·m^−1^	4.2 (0.4)^b^	83.5 (0.7)^a^	53.1 (0.3)^a^	126.4 (0.1)^a^	38.9 (0.5)^a^	0.63 (0.02)^a^	0.20 (0.01)^a^	0.82 (0.03)^a^
3.5 dS·m^−1^	5.2 (0.6)^a^	80.1 (0.6)^b^	52.6 (0.3)^a^	126.9 (0.2)^a^	37.8 (0.5)^a^	0.58 (0.02)^b^	0.18 (0.01)^b^	0.76 (0.02)^b^
Genotypes (G)								
Lastra	4.8 (0.5)^a^	80.8 (0.9)^b^	55.2 (0.2)^a^	125.4 (0.1)^b^	42.3 (0.2)^a^	0.51 (0.02)^b^	0.15 (0.01)^b^	0.66 (0.02)^b^
Green Forest	4.8 (0.5)^a^	82.9 (0.5)^a^	50.5 (0.2)^b^	127.9 (0.1)^a^	34.5 (0.3)^b^	0.70 (0.01)^a^	0.23 (0.02)^a^	0.92 (0.01)^a^
Significance^††^								
SCS	^***^	ns	^***^	^***^	^***^	^***^	^***^	^***^
EC	^*^	^***^	ns	ns	ns	^***^	^***^	^***^
G	ns	^*^	^***^	^***^	^***^	^***^	^***^	^***^
SCSxEC	^**^	^*^	ns	ns	ns	ns	ns	ns
ECxG	ns	ns	ns	ns	ns	^*^	ns	^*^
***Cichorium endivia***								
SCS^†^								
EF	2.2 (0.2)^b^	79.5 (2.8)^a^	61.5 (0.2)^a^	110.9 (0.1)^a^	24.5 (0.3)^a^	0.54 (0.02)^a^	0.15 (0.01)^a^	0.70 (0.02)^a^
FL	2.7 (0.2)^a^	81.9 (1.7)^a^	61.6 (0.1)^a^	111.1 (0.1)^a^	24.4 (0.3)^a^	0.53 (0.01)^a^	0.15 (0.01)^a^	0.69 (0.02)^a^
Salinity level (EC)								
2.5 dS·m^−1^	2.6 (0.3)^a^	79.9 (1.5)^a^	61.6 (0.2)^a^	110.9 (0.2)^a^	24.5 (0.3)^a^	0.55 (0.01)^a^	0.15 (0.01)^a^	0.71 (0.02)^a^
3.5 dS·m^−1^	2.3 (0.2)^a^	81.6 (2.9)^a^	61.5 (0.2)^a^	111.1 (0.1)^a^	24.4 (0.3)^a^	0.52 (0.02)^a^	0.15 (0.01)^a^	0.68 (0.03)^a^
Genotypes (G)								
Endive	2.2 (0.3)^b^	80.1 (2.1)^a^	60.1 (0.2)^b^	111.9 (0.1)^a^	22.4 (0.2)^b^	0.61 (0.01)^a^	0.18 (0.01)^a^	0.80 (0.02)^a^
Escarole	2.7 (0.2)^a^	81.3 (2.5)^a^	63.0 (0.1)^a^	110.1 (0.1)^a^	26.5 (0.2)^a^	0.47 (0.01)^b^	0.13 (0.01)^b^	0.60 (0.01)^b^
Significance^††^								
SCS	^*^	ns	ns	ns	ns	ns	ns	ns
EC	ns	ns	ns	ns	ns	ns	ns	ns
G	^*^	ns	^***^	^***^	^***^	^***^	^***^	^***^
ECxG	ns	ns	ns	ns	ns	^***^	^*^	^**^

† *SCS, Soilless Cultivation System; EF, Ebb and flow system; FL, Floating system*.

†† *ns*,

^*^, ^**^, and ^***^, not significant or significant at P ≤ 0.05, P ≤ 0.01, or P ≤ 0.001, respectively.

*The SCSxG, and SCSxECxG interactions in lettuce, and SCSxEC, SCSxG, and SCSxECxG interactions in C. endivia were never significant*.

a, b *Means in columns (and by effect) not sharing the same letters are significantly different according to the LSD test (α = 0.05)*.

In EF leaves, greater levels of chlorophyll *a* (CHLa), *b* (CHLb) and total (CHLtot) were detected on a fresh weight basis (Table 3). However, this seems to be due to the greater DM (Figure 2A) and SLA (Figure 2B) of these leaves (concentration-effect), as also observed by Kim et al. (2008). When the photosynthetic pigments were calculated on a dry weight basis, chlorophylls were lower in EF (11.7 vs. 13.1 μg mg^−1^ DW), implying a detrimental effect on their content by the salinity increase which occurred in this cultivation system. Concerning the leaf color, a higher hue angle (h°), which corresponds to a greater intensity of greenness and a desirable reduced yellowness, was detected in the leaves of the EF-grown plants. Moreover, they also showed a lower brightness (lower lightness index, L^*^) and colorfulness (Chroma) (Table 3), suggesting a better visual quality of these leaves compared with those from FL, attributable to the chlorophyll concentrations.

By considering the effect of the EC treatment, the highest NS salinity also impaired the biosynthesis of the chlorophyll pigments causing their reduction on a dry weight basis (11.0 vs. 14.1 μg mg DW) and but also on a fresh weight basis (Table 3), because the lower concentration-effect (Table 2) did not mask the CHL reduction. The chlorophyll changes between the two EC levels were not appreciable by instrumental measurement of the color parameters, so no significant changes in L^*^, h° or Chroma indices were detected between the two EC levels (Table 3) with no improvement in the visual quality of EC3.5 material.

Chlorophyll level can be considered a biochemical marker of salt tolerance/sensitiveness in plants with its decrease in salt-sensitive species/cultivars (Stepien and Johnson, 2009) which involves a growth reduction (Acosta-Motos et al., 2017). Probably, the slight growth decrease observed in the plants grown in EF and with the highest NS salinity level (Table 1) may be linkable to the chlorophyll impairment caused by the saline stress.

The cv. Green Forest produced a greater dry and fresh yield than “Lastra,” also showing higher area and height of leaves and specific leaf area (SLA) (Table 2). Compared with “Lastra,” “Green Forest” leaves had a more intense green color (higher h°, lower L^*^ and Chroma values) associable to its greater level of chlorophylls at both EC levels (Table 3), whereas CHLa and CHLtot content in “Lastra” were negatively affected by salinity (Supplementary Figures 1A,B). Moreover, this latter showed lower tissue hydration (lower RWC, Table 3). These data suggest that “Green Forest” is less sensitive to salinity, as also reported in other studies (Xu and Mou, 2015).

##### Cichorium endivia var. crispum and latifolium

For *C. endivia*, no significant differences emerged in terms of dry and fresh weight or of bio-physiological characteristics due to the growing system or the salinity of the NS (Table 2 and Table 3). The plants grown in FL had a higher leaf area and leaf number (Table 2) with slightly higher EL (Table 3), whereas the highest salinity slightly decreased leaf height (Table 2). Despite these negligible effects of the EC and SCSs on the morphology of the aerial part of the plant, both *C. endivia* var. *crispum* (endive) and var. *latifolium* (escarole) appeared to tolerate the saline level which occurred into the root zone more than the romaine lettuce. Little research has been performed on the response to the salinity of *C. endivia*, however, in a hydroponic study, its fresh and dry weight was negatively affected by salinity levels (6.5 and 9.8 dS m^−1^) higher than those used in our research (Kowalczyk et al., 2016). Shannon et al. (2000) have reported that endive fresh weight halved with salinity levels between 12.3 and 14.6 dS m^−1^.

By comparing *C. endivia* var. *crispum* and var. *latifolium*, endive exhibited a higher number, height and area of leaves, and less thick leaves than escarole (lower SLA) (Table 2). Nevertheless, endive had a higher level of chlorophylls, which positively affected color indices in terms of greenness intensity (lower L^*^ and Chroma) (Table 3). On the other hand, a paler color of escarole was expected as it is a characteristic of the cultivar “Bionda a cuore pieno.” In any case, escarole appears to be more salt-sensitive than endive as chlorophylls were lowered with the EC3.5 treatment (Supplementary Figures 2A–C) and in general, it showed more damaged membranes (higher EL value) (Table 3).

#### Leaf Nitrate and Antioxidant Compound Contents

##### Lactuca sativa var. longifolia

Nitrate concentration in lettuce was affected by cultivation system and EC of NS, with greater levels detected in FL and with the EC2.5 treatment (Table 4). Despite nitrate accumulation rising in plants grown in FL and under lower NS salinity, the level was in any case far below the limit imposed for lettuce by European Community Regulation 1258/2011.

**Table 4:** Effect of soilless cultivation system (SCS), salinity level (EC) of nutrient solution, and genotype (G) on nitrate, chloride and antioxidant compounds concentration of romaine lettuce (autumn cycle) and *C. endivia* (winter cycle) leaves.

**Treatments**	**Nitrate**	**Chloride**	**Vitamin C**	**Carotenoids**	**Total phenols (mg GAE^**‡**^ kg^**−1**^ FW)**
	**(mg kg**^****−1****^ **FW)**				
**Romaine lettuce**					
SCS^†^					
EF	559 (46)^b^	2010 (131)^a^	41.4 (3.1)^a^	179.9 (4.4)^a^	585.1 (32.7)^a^
FL	1188 (68)^a^	878 (55)^b^	24.7 (1.8)^b^	169.2 (5.2)^b^	497.2 (20.5)^b^
Salinity level (EC)					
2.5 dS·m^−1^	1038 (86)^a^	1125 (95)^b^	33.0 (3.5)^a^	175.3 (5.3)^a^	519.0 (22.1)^b^
3.5 dS·m^−1^	708 (75)^b^	1763 (174)^a^	33.2 (2.6)^a^	173.7 (4.5)^a^	563.4 (32.6)^a^
Genotypes (G)					
Lastra	903 (92)^a^	1487 (177)^a^	42.6 (2.9)^a^	168.3 (5.0)^b^	693.7 (21.3)^a^
Green forest	843 (82)^a^	1402 (129)^a^	23.6 (1.6)^b^	180.7 (4.6)^a^	388.7 (11.1)^b^
Significance^††^					
SCS	^**^	^***^	^***^	^*^	^***^
EC	^**^	^***^	ns	ns	^*^
G	ns	ns	^***^	^*^	^***^
SCSxEC	ns	ns	ns	ns	^***^
***Cichorium endivia***					
SCS^†^					
EF	646 (79)^b^	1784 (87)^a^	29.3 (3.0)^a^	119.6 (4.1)^a^	332.4 (12.0)^a^
FL	911 (63)^a^	1652 (139)^a^	24.5 (5.2)^a^	114.5 (3.0)^a^	313.0 (6.8)^a^
Salinity level (EC)					
2.5 dS·m^−1^	904 (63)^a^	1658 (107)^a^	30.5 (5.2)^a^	120.5 (2.5)^a^	324.7 (11.1)^a^
3.5 dS·m^−1^	653 (81)^b^	1778 (126)^a^	23.3 (2.7)^a^	114.2 (5.9)^a^	320.7 (8.6)^a^
Genotypes (G)					
Endive	776 (83)^a^	1555 (109)^b^	24.6 (4.4)^a^	133.6 (2.9)^a^	333.9 (9.2)^a^
Escarole	781 (81)^a^	1881 (105)^a^	29.2 (4.1)^a^	102.1 (2.3)^b^	311.5 (10.1)^a^
Significance^††^					
SCS	^**^	ns	ns	ns	ns
EC	^**^	ns	ns	ns	ns
G	ns	^*^	ns	^***^	ns

† *SCS, Soilless Cultivation System; EF, Ebb and flow system; FL, Floating system*.

†† *NS*,

^*^, ^**^, and ^***^, not significant or significant at P ≤ 0.05, P ≤ 0.01, or P ≤ 0.001, respectively. The SCSxG, ECxG, and SCSxECxG interactions in lettuce, and SCSxEC, SCSxG, ECxG and SCSxECxG interactions in C. endivia were never significant.

a, b *Means in columns (and by effect) not sharing the same letters are significantly different according to LSD test (α = 0.05*.

‡ *GAE, gallic acid equivalent*.

**Figure 4:**
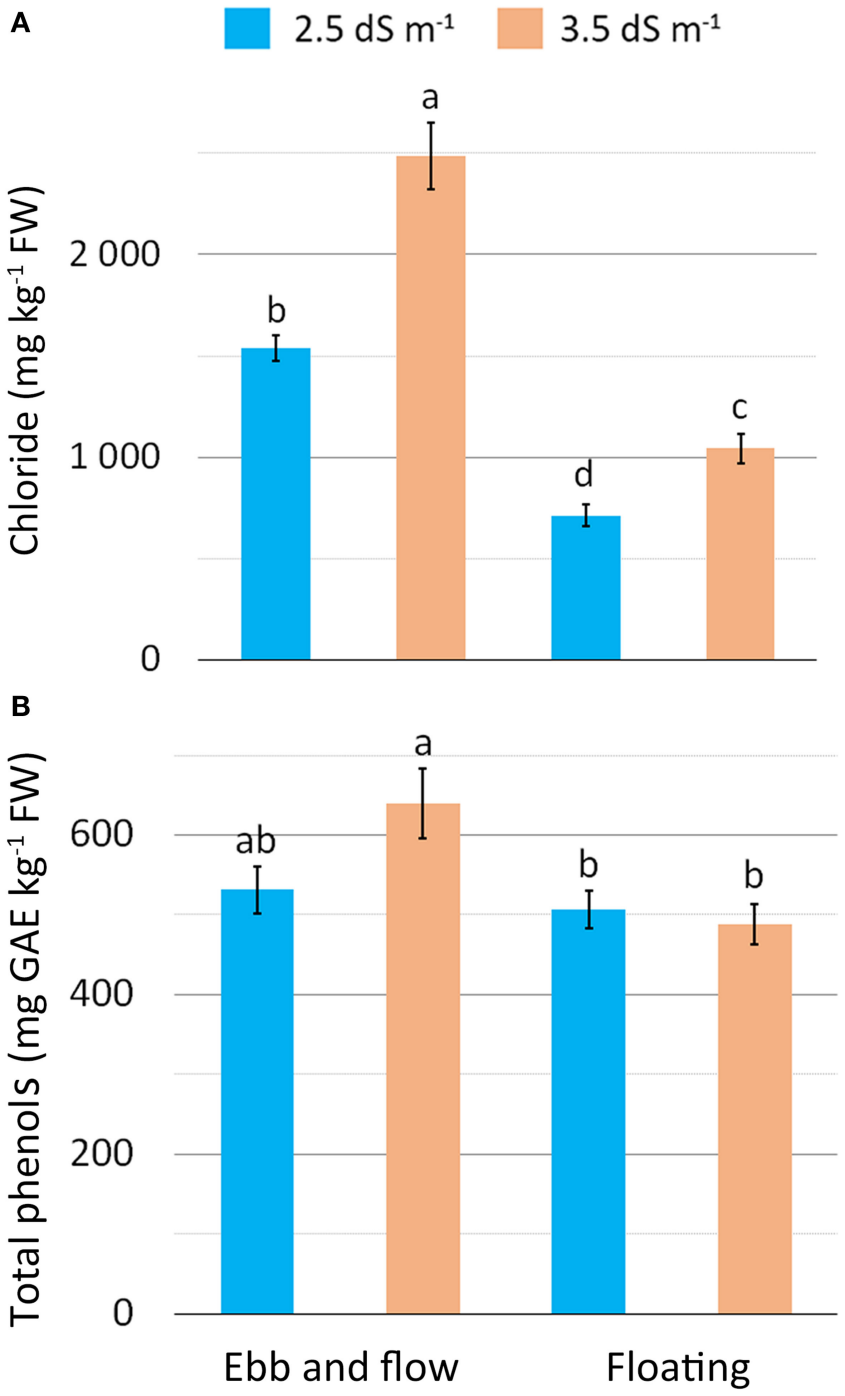
Effect of soilless cultivation system and nutrient solution electrical conductivity on chloride **(A)** and total phenol **(B)** concentration in romaine lettuce grown in a greenhouse during the autumn. Vertical bars indicate ±SE of mean (*n* = 9) of the observed values, with different letters significantly different according to the LSD test (α = 0.05).

Chloride concentration was the highest in EF and EC3.5 (Table 4), and especially in the EF/EC3.5 combination (Figure 4A), confirming the expected accumulation of Cl^−^ in plants exposed to saline (NaCl) stress (Wu and Li, 2019). Chloride showed an opposite trend to nitrate as it is well-known that salinity can reduce nitrate accumulation in leafy vegetables due to antagonism between nitrate and chloride for the same root anion channel (Bian et al., 2020). A linear decrease in nitrate concentration has been reported in romaine lettuce baby-leaf grown in FL with an increase in NS salinity up to 4.8 dS m^−1^ (Scuderi et al., 2011), in agreement with other experiments on leafy vegetables (Barbieri et al., 2011). The increase in EC from 2.5 to 3.5 dS m^−1^ resulted in a reduction in nitrate concentration along with a Cl^−^ rise in soilless-grown wild rocket (Bonasia et al., 2017).

Despite regulating leaf osmotic potential and turgor as well as stimulating plant growth, chloride may exert negative effects at higher levels than critical toxicity values (Colmenero-Flores et al., 2019). Therefore, the general rise in chloride concentration in leaves grown in EF and with the highest saline treatment could have caused the slight decrease in plant dry biomass accumulation (Table 2) by inhibiting nitrate accumulation (Table 4) (Colmenero-Flores et al., 2019) and/or negatively affecting chlorophylls as high Cl^−^ concentration may reduce the photosynthetic capacity and quantum yield due to chlorophyll degradation (Tavakkoli et al., 2010). Moreover, it is known that chloride ions inhibit the activity of the enzymes involved in the N metabolism, such as NR, NiR, GS, and GDH, and consequently N assimilation (Barber et al., 1989; Debouba et al., 2006, 2007).

With the EF system, vitamin C, total phenols (TP) and carotenoid concentration rose compared to FL (Table 4). In contrast, no changes in these compounds were detected between EC2.5 and EC3.5 plants except for the EF/EC3.5 combination when TP were the highest (Figure 4B). These compounds of secondary metabolism act as antioxidants to remove reactive oxygen species (ROS) (De Pascale et al., 2001; Taârit et al., 2012) produced as a consequence of biotic and abiotic stress, such as salinity (Acosta-Motos et al., 2017). A similar response in terms of antioxidant compound improvement has also been reported for carotenoids and phenols in romaine lettuce (Kim et al., 2008; Mahmoudi et al., 2010) and other vegetables with higher vitamin C in tomato and *Cichorium spinosum* (De Pascale et al., 2001; Petropoulos et al., 2017), total phenols in sage, radish and broccoli (Yuan et al., 2010; Taârit et al., 2012; Guo et al., 2014), and carotenoids in tomato (De Pascale et al., 2001) associated with a rise in the EC level in the cultivation medium. In general, the enhancement in antioxidant compounds is related to a mild/moderate saline stress, when this salinity species/cultivar-specific threshold is exceeded reduced antioxidant compounds have been reported (Rouphael and Kyriacou, 2018) as presumably, the antioxidant system does not effectively support ROS scavenging (Bonasia et al., 2017).

In the present work, salt-stress appears more pronounced in EF-grown plants as it triggered a biochemical response to counteract the oxidant toxic molecules, with TP synthesis promoted when plants experienced a higher salinity load (EF/EC3.5). These results are strictly consistent with the physiological status of tissues (Figures 3A,B) and can be related to chloride concentration (Figure 4A).

Based on all the above considerations, also taking into account the low impact on plant growth and chlorophylls, the saline stress imposed in this study with EC3.5, EF and their combination can be considered to range from mild to moderate for romaine lettuce.

In addition to their role as a defense system, many secondary metabolites are well-known as nutritional bioactive compounds with beneficial health-related properties, such as anticancer, antioxidant, and anti-inflammatory activities (Kim et al., 2008). Therefore, the saline stress at the level applied in this research with the EF system (moderate) enhanced the nutritional value of romaine lettuce.

No differences in nitrate content emerged between cultivars. In “Lastra” both vitamin C and TP were two-fold higher than in “Green Forest,” which showed a slightly greater carotenoid concentration (Table 4), confirming that “Lastra” was more sensitive to saline stress.

##### Cichorium endivia var. crispum and latifolium

Endive, like lettuce, is classified among the “high nitrate content” vegetables and it can easily accumulate more than 2,500 mg kg^−1^ FW of this ion (Santamaria, 2006).

The EF system and the EC3.5 treatment were confirmed to reduce nitrate concentration in the tested *C. endivia* botanical varieties. However, the decrease (−46 and −38% in EF and EC3.5, respectively) was less evident than in lettuce (−112% in EF and −47% EC3.5). In *C. endivia*, in contrast to lettuce, no change in chloride concentration was detected as affected by SCS and NS EC. The fact that this species did not accumulate chloride in EF and EC3.5 leaves can be related to its higher salt-tolerance, as an efficient exclusion of Cl^−^ from either roots or shoots, avoiding the excessive accumulation of Cl^−^ in plant tissues, is reported to be important for the overall plant salt tolerance (Wu and Li, 2019). Another implication of these results is that the observed reduction in leaf nitrate concentration in EF and EC3.5 plants may be due to the replacement of this anion as an osmolyte by compounds other than Cl^−^. Among inorganic anions, neither the phosphate nor the sulfate concentration changed according to NS EC (respectively 280 and 646 mg kg^−1^ FW, on average in EC2.5 and EC3.5) and SCS (respectively 280 and 649 mg kg^−1^ FW, on average in FL and EF) so a compound such as proline could have been involved in the osmotic adjustment as also observed in *C. endivia* var. *crispum* (Kowalczyk et al., 2016) and other species (Zhu et al., 2008; Hajlaoui et al., 2010). The unchanged concentration of the considered antioxidant compounds in the SCSs and EC treatments (Table 4) confirms that plants did not experience salt-stress. However, *C. endivia* response to salinity has been little studied and it deserves further research to understand the specific mechanisms of this species to cope with salinity.

Endive and escarole did not differ in terms of nitrate, vitamin C or total phenol concentration, but escarole showed a higher concentration of chloride and a lower concentration of carotenoids (Table 4).

### Effects of Nutrient Solution Withdrawal, Soilless Cultivation System and Genotypes (Experiment 2)

This experiment was performed on plants grown in EF and FL systems at 2.5 dS m^−1^ EC level to test the replacement of NS with freshwater before the harvest (NS withdrawal -WD) as a strategy to reduce leaf nitrate concentration. We decided to apply this treatment at the lower NS salinity in autumn and winter cycles characterized by the lower solar radiation as these are the cultivation and sunlight conditions (Bian et al., 2020) that favor nitrate accumulation.

#### Growth, Yield, Leaf Bio-Physiological Traits, Nitrate and Antioxidant Compound Contents

The significance of the F test for NS management, SCS, genotype and their interactions for lettuce and *C. endivia* are reported in Table 4. As the main and interaction effects of SGSs and genotypes have previously been considered, in this section we only focused on the main effect of NS management and its interaction with both SCS and genotypes.

##### Lactuca sativa var. longifolia

Lettuce growth (dry weight) was not affected by the NS replacement with freshwater (WD treatment) (Table 5). However, a higher dry matter concentration was observed when plants were grown in water for 3 days before harvest especially in EF, so provoking a reduction in fresh yield in this SCS (Table 6).

**Table 5:** The significance of the ANOVA F test for the effects of soilless cultivation system (SCS), nutrient solution management before harvest (NSM) and genotype (G), and their interaction, on yield, bio-morpho-physiological traits, nitrate, chloride and antioxidant compounds concentration of romaine lettuce and *C. endivia* leaves.

**Treatments**	**Fresh weight**	**Dry weight**	**Dry mass**	${\text{NO}}_{3}^{-}$ **(FW or DW basis)**	**Cl^**−**^ (FW or DW basis)**	**Fresh weight**	**Dry weight**	**Dry mass**	**Leaf area**	**Leaf height**	**Leaf number**	**Specific leaf area**	**Chlorophylls (FW basis)**	**Chlorophylls (DW basis)**	L^*^	**h^**°**^**	**Chroma**	**Electrolyte leakage**	**Relative water content**	**Total phenols (FW or DW basis)**	**Vitamin C (FW basis)**	**Vitamin C (DW basis)**	**Carotenoids (FW or DW basis)**	${\text{NO}}_{3}^{-}$ **(FW or DW basis)**	**Cl^**−**^ (FW or DW basis)**
	**Romaine lettuce**					***Cichorium endivia***																			
SCS^†^	^***^^§^	^**^	^***^	^***^	^***^	ns	ns	^*^	^***^	^***^	^***^	ns	ns	ns	ns	ns	ns	^***^	ns	^***^	ns	ns	ns	ns	^**^
NSM^††^	^*^	ns	^***^	^***^	^***^	^*^	ns	^**^	^*^	^***^	ns	ns	ns	^*^	ns	^***^	^**^	^*^	ns	^**^	ns	^*^	ns	^***^	ns
Genotype (G)	^***^	^***^	ns	ns	ns	ns	ns	^**^	^**^	ns	^***^	^***^	^***^	^***^	^***^	^***^	^***^	^*^	^*^	ns	ns	ns	^***^	ns	ns
SCSxNSM	^**^	ns	^*^	^*^	^*^	ns	ns	ns	ns	ns	ns	ns	ns	ns	ns	ns	ns	ns	ns	ns	ns	ns	ns	ns	ns
SCS*G	ns	ns	ns	ns	ns	ns	^*^	ns	ns	^*^	ns	ns	ns	ns	ns	ns	ns	ns	ns	ns	ns	ns	ns	ns	ns

† *SCS, Soilless Cultivation System; EF, Ebb and flow system; FL, Floating system*.

†† *NSM is nutrient solution management: nutrient solution replacement (WD) or not (noWD) with freshwater 3 (lettuce) or 5 (C. endivia) days before harvest*.

§ *ns*,

^*^, ^**^, and ^***^, not significant at P ≤ 0.05, P ≤ 0.01, or P ≤ 0.001, respectively. In both species, the NSMxG, and SCSxGxNSM interactions were never significant.

**Table 6:** Effect of soilless cultivation system and nutrient solution management before harvest on growth, productivity, and nitrate and chloride concentration of romaine lettuce (autumn cycle) leaves, with the standard error of the mean in brackets.

**Soiless cultivation system**	**NSM^***††***^**	**Fresh weight (kg m^**−2**^)**	**Dry mass (g kg^**−1**^ FW)**	**Nitrate**	**Chloride**
				**(mg kg**^****−1****^ **FW)**	
EF^†^	noWD	1.9 (0.1)^a^	47.8 (1.1)a^b^	731 (33)^b^	1537 (216)^b^
	WD	1.3 (0.1)^b^	63.0 (1.9)^a^	320 (35)^c^	2473 (486)^a^
FL	noWD	2.2 (0.2)^a^	42.4 (0.9)^c^	1345 (112)^a^	714 (189)^c^
	WD	2.3 (0.2)^a^	45.8 (1.6)^c^^b^	694 (64)^b^	1276 (311)^b^

† *EF, Ebb and flow system; FL, Floating system*.

†† *NSM is nutrient solution management: nutrient solution replacement (WD) or not (noWD) with freshwater 3 (lettuce) or 5 (C. endivia) days before harvest*.

a−c *Means in columns not sharing the same letters are significantly different according to the LSD test (α = 0.05)*.

Nitrate concentration significantly decreased in WD plants compared with the noWD ones whereas a significant opposite behavior was observed for chloride concentration (Table 6). It is well-known that during a period of deprivation, nitrates stored in vacuoles are used to sustain plant growth (Bian et al., 2020) and chloride may replace ${\text{NO}}_{3}^{-}$ as osmolyte, as reported for endive (Santamaria and Elia, 1997), chicory and rocket (Santamaria et al., 1998), especially when Cl^−^ is added to NS (pak-choi, Zhu et al., 1997; cultivated cardoon, Borgognone et al., 2016; basil, Corrado et al., 2020) since a very low ${\text{NO}}_{3}^{-}$:Cl^−^ ratio promotes Cl^−^ uptake (Colmenero-Flores et al., 2019).

In this study, in FL-grown plants, the withdrawal of NS resulted in a decrease in nitrate level with no effects on yield (Table 6), in agreement with findings obtained both in lamb's lettuce (Gonnella et al., 2004) in the same soilless system. On the contrary, in the EF system, where a lower nitrate accumulation was proved due to the rise in salinity, the NS withdrawal caused a drop in ${\text{NO}}_{3}^{-}$ to a very low level (Table 6) leading us to suppose that sub-optimal N conditions occurred. In chicory and rocket (Santamaria et al., 1998), in lamb's lettuce (Gonnella et al., 2004) and cultivated cardoon (Borgognone et al., 2016) nitrogen deprivation resulted in a decrease in both nitrate and total N.

It is known that in many crops N availability below optimal level starts several physiological adjustments aimed at maintaining cellular N concentration (specific leaf N) at the threshold to sustain photosynthetic machinery and dry matter production. These adjustments involve a DM concentration at the expense of leaf expansion (Gastal et al., 2015). In our study, leaf DM was the highest in EF/WD lettuce (Table 6) and, although we did not measure morphological leaf traits, the observed lowering of fresh yield in the EF/WD combination may be assumed to be imputable to a reduction in leaf growth. In agreement with our results, in NFT-lettuce deprived of NS for 10–6 days before harvest, Tabaglio et al. (2020) observed a decline in fresh yield which was related to the DM enhancement.

The general improvement of DM obtained as a consequence of the water-treatment may be considered a positive effect for baby-leaf vegetables as it enhances their suitability to processing (Clarkson et al., 2003; Conversa et al., 2014).

No significant interaction between NS management and Genotype was detected in fresh and dry weight, DM, nitrate or chloride concentration (Table 5) suggesting that both “Green Forest” and “Lastra” reacted similarly to the withdrawal of NS before harvest.

##### Cichorium endivia var. crispum and latifolium

No significant interaction NSM x SCS and NSM x G was detected for this species. Similarly to lettuce, in *C. endivia* no significant reduction in dry yield occurred with the WD treatment. Irrespectively of the SCS, fresh yield decreased, probably due to a concentration effect as DM rose by 10%. On the other hand, with WD treatment, a significant reduction in nitrate concentration occurred (Table 7).

**Table 7:** Effect of nutrient solution management before harvest on yield, bio-morpho-physiological traits, nitrate, chloride and antioxidant compound concentrations of *C. endivia* leaves. The standard error of mean is in brackets.

**Parameter**	**Nutrient solution management**^**†**^	
	**WD**	**noWD**
Fresh weight (kg m^−2^)	1.7 (0.1)^b^	2.2 (0.1)^a^
Dry mass (g kg^−1^ FW)	62.1 (0.2)^a^	56.2 (0.1)^b^
Leaf area (cm^−2^)	33.7 (1.1)^b^	37.6 (2.4)^a^
Leaf height (cm)	11.2 (0.1)^b^	13.7 (0.1) a
Chlorophylls (μg mg^−1^ DW)	11.0 (0.1)^b^	12.5 (0.1)^a^
h°	111.4 (0.2)^a^	110.9 (0.2)^b^
Chroma	23.9 (0.3)^b^	24.5 (0.3)^a^
Nitrate (mg kg^−1^ FW)	396 (31)^b^	904 (63)^a^
Total phenols (mg GAEs^‡^ g^−1^ DW)	0.61 (0.12)^a^	0.58 (0.17)^b^
Vitamin C (mg kg^−1^ DW)	12.4 (3.9)^a^	10.6 (5.2)^b^

† *WD, nutrient solution replacement with freshwater 3 (lettuce) or 5 (C. endivia) days before harvest. noWD, with no nutrient solution replacement*.

a, b *Means in rows not sharing the same letters are significantly different according to the LSD test (α = 0.05)*.

‡ *GAE, gallic acid equivalent*.

As for lettuce, in *C. endivia* these responses can be argued as a physiological adjustment to the suboptimal N condition imposed by the withdrawal of NS. Probably, the prolonged NS deprivation in this species (6 days vs. 3 days in lettuce) canceled out the differences in responses between SCSs, which in contrast were observed in lettuce (Table 6).

For *C. endivia*, the morpho-physiological and the antioxidative leaf traits were also measured. Both the leaf area and height were significantly reduced in WD compared with noWD treatment (Table 7). The SLA, chlorophyll concentrations on a fresh weight basis were not affected by the NS replacement with water (Table 5), even if a slight reduction occurred for chlorophylls measured on a dry weight basis (Table 7) proving that plants were slightly suffering from N starvation. However, color traits were improved in WD leaves due to the rise in h° and the lower Chroma (Table 7). No difference in physiological tissue status was detected with the replacement of NS with water, nor in carotenoid level. In contrast, the concentration of vitamin C and TP (Table 7) increased in agreement with other authors (Borgognone et al., 2016). These latter related the improvement in TP in cultivated cardoon to the N-limiting stress which occurred under N-deprivation treatment.

In disagreement with our results, the supply for 7 days before the harvest with an NS deprived of 90% of the initial N concentration, affected neither dry nor fresh yield in endive heads (Santamaria et al., 1997). However, despite a reduction in the nitrate concentration having been reported, the level of this anion was much higher (1,504 mg kg^−1^ FW) than that in our trial, leading us to suppose that no sub-optimal nitrogen level occurred.

Contrary to lettuce, chloride did not show changes between noWD and WD plants to counteract nitrate depletion (Table 5), confirming that no relationship can be supposed in *C. endivia* between the level of these anions. However, in endive when nitrate decrease was due to ${\text{NH}}_{4}^{+}$:${\text{NO}}_{3}^{-}$ ratios (70:30 and 100:0) in the NS higher than that used in the present studies (10:90) a rise in chloride uptake was evident, suggesting a role of ${\text{NH}}_{4}^{+}$ in Cl^−^ uptake (Santamaria and Elia, 1997). In lamb's lettuce, no changes in Cl^−^ concentration after 3 days in water were reported, despite a nitrate reduction (Gonnella et al., 2004).

### Effects of the Growing Season, Soilless Cultivation System and Genotype (Experiment 3)

Lettuce and endive/escarole are considered cool-season crops which thrive in areas where the mean temperatures range between 15 and 18°C (Alvino and Barbieri, 2016). Nevertheless, over the whole growing season (autumn-spring), varying temperature regimes and radiation levels may occur as repeated crop cycles can be carried out during this period. It is well-known that temperatures along with solar radiation affect nitrate accumulation, plant growth, yield and product quality in interaction with the growing conditions and genotypes (Fallovo et al., 2009; Sublett et al., 2018), so this trial was performed to compare two crop cycles for romaine lettuce cultivars (autumn vs. early-spring) and *C. endivia* varieties (winter vs. late-spring) (Table 1) both grown in EF and FL at EC3.5.

#### Growth, Yield, Leaf Bio-Physiological, Nutritional and Antioxidative Traits

The significance of the F test for the crop cycle (CC), SCS, genotypes and their interactions for lettuce and *C. endivia* is reported in Table 8. As the main effects of SCS, genotypes and their interactions have been considered above, in this section, we focus on the main effect of the crop cycle and its interaction with both SCS and genotypes.

**Table 8:** The significance of F test of ANOVA for the effects of the crop cycle (CC), soilless cultivation system (SCS), and genotype (G), and their interactions, on yield, bio-morpho-physiological traits, nitrate, chloride and antioxidant compound concentration in romaine lettuce and *C. endivia*.

**Treatments**	**Fresh weight**	**Dry weight**	**Dry mass**	**Leaf area**	**Leaf heght**	**Leaf number**	**Specific leaf area**	**Chlorophyll a (FW basis)**	**Chlorophyll b(FW basis)**	**Total chlorophylls (FW basis)**	L^*^	**h^**°**^**	**Crhoma**	**Electrolyte leakage**	**Relative water content**	**Total phenols (FW or DW basis)**	**Carotenoids (FW basis)**	${\text{NO}}_{3}^{-}$ **(DW basis)**	${\text{NO}}_{3}^{-}$ **(FW basis)**	**Cl^**−**^ (FW or DW basis)**
**Romaine lettuce**																				
Crop cycle (CC)	^*^^§^	^***^	^***^	^***^	^***^	^***^	^***^	^*^	^***^	^**^	^***^	^***^	^***^	^***^	^***^	^***^	^***^	^***^	^***^	^***^
SCS^†^	^***^	^***^	^***^	^***^	^***^	^***^	^**^	^*^	ns	^*^	^**^	^***^	^***^	^***^	^***^	^*^	ns	^***^	^***^	^***^
Genotype (G)	^***^	^***^	ns	ns	^*^	^***^	ns	^***^	^***^	^***^	^***^	^***^	^***^	ns	^*^	^***^	^***^	ns	^*^	ns
CCxSCS	^*^	^*^	^**^	^***^	^**^	ns	^*^	ns	^**^	ns	^**^	^***^	^***^	^***^	ns	^***^	^*^	^***^	^*^	^*^
CCxG	^***^	^***^	ns	ns	^***^	^***^	^***^	^***^	^***^	^***^	ns	^*^	ns	ns	ns	ns	ns	ns	ns	ns
SGSxG	^***^	ns	ns	^*^	^***^	ns	ns	ns	ns	ns	ns	ns	ns	ns	ns	ns	^***^	ns	ns	ns
***Cichorium endivia***																				
Crop cycle (CC)	^**^	^***^	^***^	^***^	^***^	ns	^***^	^***^	^**^	^***^	^***^	^***^	^***^	^***^	^*^	^***^	^***^	^***^	^***^	^***^
SCS^†^	ns	ns	^**^	ns	^***^	ns	ns	ns	ns	ns	^**^	^**^	^*^	^**^	ns	ns	ns	^*^	^**^	ns
Genotype (G)	ns	ns	^**^	ns	^***^	^***^	^*^	^*^	ns	^*^	ns	ns	ns	^*^	ns	ns	^***^	ns	ns	ns
CCxSCS	ns	ns	^***^	ns	^***^	^*^	^**^	ns	ns	ns	^***^	^***^	^***^	ns	ns	^**^	ns	^**^	^*^	^*^
CCxG	ns	ns	ns	ns	ns	ns	ns	ns	ns	ns	ns	ns	ns	ns	ns	^**^	^***^	ns	ns	ns
SGSxG	ns	ns	ns	ns	^**^	ns	ns	^***^	ns	^**^	ns	ns	ns	^**^	^*^	ns	ns	ns	ns	ns

§ *ns*,

^*^, ^**^, and ^***^, not significant at P ≤ 0.05, P ≤ 0.01, or P ≤ 0.001, respectively. In both species, the CCxSCSxG interaction was never significant.

† *SCS, Soilless Cultivation System; EF, Ebb and flow system; FL, Floating system*.

##### Lactuca sativa var. longifolia

Significant CC x SCS interactions were detected for many productive and qualitative parameters (Table 8). The dry weight showed an increasing trend passing from the autumn to early-spring (ES) cycle, specifically in the FL system, where leaves also showed a higher leaf area (Table 9). In general, compared with the autumn cycle, the early-spring grown plants produced more leaves (4.8 vs. 4.2) with lower water content (RWC) (80.1 vs. 76.2%) and they were tighter and thicker due to the enhancement in DM and SLA (Table 9). Mean values of NS EC and oxygen concentration measured during both crop cycles did not show substantial changes passing from the autumn to early-spring season (Supplementary Table 1), so these parameters are not involved in the plant seasonal response.

**Table 9:** Effect of the crop cycle and soilless cultivation system (SCS) on yield, bio-morpho-physiological traits, nitrate, chloride and antioxidant compound concentrations of romaine lettuce (autumn/early-spring cycles) and *C. endivia* (winter/late-spring cycles) leaves, with the standard error of mean in brackets.

**Treatments**															
**Crop cycle**	**SCS^**†**^**	**Fresh yield (kg m^**−2**^)**	**Dry yield (g m^**−2**^)**	**Dry mass (g kg^**−1**^ FW)**	**Leaf area (cm^**−2**^)**	**Leaf height (cm)**	**Specific leaf area (g cm^**−2**^)**	L^*^	**h^**°**^**	**Chroma**	**Electrolyte leakage (%)**	**Total phenol (mg GAE^**‡**^ kg^**−1**^ FW)**	**Carotenoids (mg kg^**−1**^ FW)**	**Nitrate (mg kg^**−1**^ FW)**	**Chloride (mg kg^**−1**^ FW)**
**Romaine lettuce**															
Autumn	EF	1.3 ^b^ (0.1)	81 ^b^ (6.8)	59.0 ^b^ (2.1)	33.4 ^b^ (1.1)	13.6 ^b^ (0.2)	1.9 ^b^ (0.10)	51.5 ^b^ (0.4)	127.6 ^b^ (0.2)	35.8 ^b^ (0.6)	7.6 ^a^ (0.4)	639 ^a^ (53)	0.18 ^a^ (0.01)	386 ^c^ (48)	2484 ^b^ (165)
	FL	2.0 ^a^ (0.2)	87 ^a^^b^ (6.0)	45.1 ^c^ (7.0)	34.8 ^b^ (1.3)	15.1 ^a^ (0.2)	1.5 ^c^ (0.03)	53.7 ^a^ (0.4)	126.2 ^c^ (0.2)	39.8 ^a^ (0.6)	2.7 ^b^ (0.1)	488 ^a^^b^ (32)	0.17 ^a^ (0.01)	1031 ^a^ (45)	1043 ^c^ (70)
Early-spring	EF	1.3 ^b^ (0.1)	87 ^a^^b^ (3.1)	65.8 ^a^ (2.0)	34.6 ^b^ (2.8)	11.1 ^c^ (0.3)	2.6 ^a^ (0.08)	50.0 ^c^^b^ (1.0)	131.4 ^a^ (0.3)	32.3 ^c^ (0.8)	1.8 ^c^^b^ (0.2)	400 ^b^ (31)	0.12 ^b^ (0.02)	350 ^c^ (35)	4145 ^a^ (309)
	FL	1.7 ^a^^b^ (0.1)	106 ^a^ (3.1)	60.1 ^a^^b^ (0.9)	50.0 ^a^ (2.6)	13.5 ^b^ (0.2)	2.6 ^a^ (0.10)	49.8 ^c^ (0.6)	132.1 ^a^ (0.4)	31.7 ^c^ (0.8)	1.6 ^c^ (0.1)	473 ^b^ (72)	0.15 ^a^^b^ (0.03)	717 ^b^ (80)	2921 ^b^ (248)
***Cichorium endivia***															
Winter	EF	1.9 ^b^ (0.2)	102 ^b^ (8.7)	55.3 ^b^ (0.9)	33.3 ^b^ (1.8)	16.7 ^a^ (0.2)	2.1 ^b^ (0.1)	61.3 ^a^ (0.4)	111.1 ^a^ (0.2)	24.1 ^c^ (0.5)	2.2 ^a^ (0.3)	332 ^a^ (12)	0.12 ^b^ (0.01)	431 ^c^ (47)	1733 ^c^ (122)
	FL	2.1 ^b^ (0.1)	111 ^b^ (16.4)	55.4 ^b^ (1.1)	35.7 ^b^ (2.1)	15.1 ^b^ (0.2)	1.9 ^b^ (0.2)	61.6 ^a^ (0.5)	111.1 ^a^ (0.3)	24.6 ^c^ (0.7)	2.4 ^a^ (0.2)	313 ^a^^b^ (7)	0.11 ^b^ (0.01)	875 ^b^ (85)	1823 ^b^^c^ (232)
Late-spring	EF	2.6 ^a^ (0.1)	146 ^a^ (7.3)	55.0 ^b^ (1.8)	49.5 ^a^ (3.2)	12.8 ^c^ (0.3)	2.2 ^b^ (0.1)	57.6 ^b^ (0.3)	110.0 ^b^ (0.4)	34.1 ^a^ (0.4)	0.5 ^b^ (0.1)	251 ^c^ (14)	1.95 ^a^ (0.14)	1292 ^a^ (84)	2243 ^b^ (156)
	FL	2.4 ^a^ (0.2)	158 ^a^ (6.9)	65.8 ^a^ (3.0)	48.8 ^a^ (2.8)	13.2 ^c^ (0.2)	2.6 ^a^ (0.1)	55.7 ^c^ (0.4)	110.9 ^a^ (0.2)	32.3 ^b^ (0.4)	1.1 ^b^ (0.1)	294 ^b^ (11)	1.87 ^a^ (0.21)	1498 ^a^ (160)	3162 ^a^ (295)

† *SCS is Soilless Cultivation System; EF, Ebb and flow system; FL, Floating system*.

‡ *GAE, gallic acid equivalent*.

a−c *Means in columns not sharing the same letters are significantly different according to the LSD test (α = 0.05)*.

During the early-spring cycle, temperatures in the greenhouse were lower than in the autumn cycle despite higher solar radiation (Figure 1). Probably, the temperatures were closer to the optimal ones along with the longer photoperiod in the early-spring crop, slightly prompting plant growth and especially involved some physiological adjustments and morphological changes leading to more compact plants and firmer leaves. Similar results are reported by other authors who found higher growth (Fallovo et al., 2009; Sublett et al., 2018) and DM (Sublett et al., 2018) in loose-leaf lettuce produced under higher solar radiation as well as in butterhead lettuce leaves (Bonasia et al., 2013) and baby-leaf wild rocket (Bonasia et al., 2017). In the ES period, the FL-grown plants showed the largest increase in DM and SLA resulting in a reduction in their fresh yield (Table 9) compared with the autumn crop. However, despite this slight yield decline, baby-leaf lettuce obtained in FL in the early-spring cycle may be considered to have improved post-harvest processability (Clarkson et al., 2003; Conversa et al., 2014) compared to those obtained under autumn climate which appeared to have worse post-harvest handling resistance (taller and thinner). No seasonal changes in lettuce yield were observed in EF, confirming the trend to produce less than in FL at EC3.5 (Table 2; Figure 2C).

In terms of visual quality, romaine lettuce leaves were better in appearance when grown under early-spring climate as they showed a reduction in the brightness (lower L^*^ and Chroma) along with an improvement in greenness (higher h°) (Table 9), which was due to an enhancement in the level of chlorophylls (0.87 vs. 0.76 μg mg^−1^ FW) associable to a concentration effect. This color change was more evident in FL plants contributing to enhancing the quality of this product compared to the autumn one. A similar trend in chlorophyll content and color of baby-leaf lettuce was also detected by Fallovo et al. (2009) with increasing seasonal solar radiation. Additionally, the early-spring plants appear to be grown under less stressing conditions as leaves had less damaged membranes (much lower EL) and weakened antioxidative status (lower TP and carotenoids concentration) (Table 9).

With the FL system, nitrate accumulation was always higher than EF and as expected, it decreased in the early-spring conditions due to higher radiation availability (Blom-Zandstra, 1989). Accordingly, a rise in chloride was observed confirming its well-known replacement in vacuoles of ${\text{NO}}_{3}^{-}$ (Bian et al., 2020) used for sustaining the higher plant growth (Table 9).

Very low accumulation of ${\text{NO}}_{3}^{-}$ was observed in EF-grown plants with no differences between the autumn and early-spring cycles, whereas Cl^−^ in EF/early-spring plants hugely increased reaching the greatest level (Table 9) among all the trials performed on lettuce (Figure 4A, Table 5) highlighting a greater rise in salinity. The highest Cl^−^ concentration occurring in early-spring EF-plants could have exhibited toxics effects, altering the nitrogen metabolism, inhibiting the activity of the nitrate reductase enzyme (Barber et al., 1989). Hence, it resulted in neither a reduction in nitrate content nor growth improvement of these plants, even though higher radiation should have positively affected nitrogen assimilation. Similar results have been obtained for EF- and Fl-grown wild rocket (Bonasia et al., 2017).

The Green Forest cultivar was affected by the climate variability as it showed a reduction in yield in the early-spring season essentially due to the lower leaf height. On the other hand, both in “Green Forest” and “Lastra” the visual quality was improved under the early-spring conditions with an enhancement in chlorophylls and h° (Supplementary Table 2).

##### Cichorium endivia var. crispum and latifolium

The late-spring (LS) cycle was characterized by both higher temperatures and greater solar radiation compared to the winter cycle (Figure 1). The LS climate improved both fresh and dry weight in *C. endivia* because of the enhancement of leaf area, despite the decline in leaf height (larger leaves), particularly in EF (Tables 8, 9). As for lettuce, a scarce variability in mean values of NS EC and oxygen concentration measured during crop cycles was observed passing from the winter to late-spring season (Supplementary Table 1) so the plant seasonal response can only be attributable to the climate.

Chlorophylls decreased from 0.68 to 0.46 μg mg^−1^ FW in the winter and late-spring season, respectively, however leaf appearance did not show substantial changes with a slightly lower visual quality in the LS cycle especially in EF leaves, due to a reduction in greenness (lower h°) along with a higher brightness (higher C) (Table 9). Carotenoids acting as photo protectors were massively increased in late-spring leaves which also had less damaged membranes (lower EL) with lower TP concentration underlining less stressed tissues (Table 9). However, they had a lower RWC (74.4 vs. 80.7%).

Overall, these findings underline that climate strongly affected the growth and productivity of *C. endivia*, with the most favorable condition being at a daily mean temperature of 20°C and high radiation. Whereas, except for the above described slight effects of EF on visual quality, the growing systems poorly influenced *C. endivia* performance.

Surprisingly, nitrate concentration rose in the May-June cycle (Table 9) especially in EF (by three-fold) canceling the difference between the growing systems observed during the winter cycle (Table 9). Probably late-spring temperatures were highly effective in enhancing uptake of this ion as also reported for soilless-grown lettuce by Fallovo et al. (2009) and they were much more important than the radiation and the salinity effect deriving from the SGSs. On the contrary, in the winter cycle, low temperatures under weak light conditions seemed to impair nitrate uptake and translocation as reported in Bian et al. (2020). The late-spring temperature also prompts Cl^−^ uptake, especially in FL (Table 9) confirming the lack of an inverse correlation between nitrate and chloride in *C. endivia*.

The differences in yield, bio-morphological, bio-physiological and antioxidative traits between endive and escarole observed in the winter cycle (Tables 2–4) were substantially confirmed under late-spring conditions (Table 8) except for the greater level of total phenols (293 vs. 252 mg GAE kg^−1^ FW) and carotenoids (2.2 vs. 1.4 mg kg^−1^ FW) shown by escarole under the more favorable conditions.

## Conclusions

The effectiveness of the strategies considered in this work to reduce nitrate level in soilless grown baby-leaf romaine lettuce and *C. endivia* mainly depends on species-specific responses to salinity imposed by the soilless system and nutrient solution, and then on the climatic conditions throughout the growing season.

In the salt-sensitive lettuce, with the EF system or nutrient solution EC at 3.5 dS m^−1^, ${\text{NO}}_{3}^{-}$ concentration may be reduced, allowing the productivity to be maintained at a typical level for the crop cycle. Under less favorable climatic conditions (higher temperatures and lower solar radiation of an autumn cycle) the EF system should be preferred as it produces baby-leaf lettuce with improved color, thickness and antioxidative/nutritional properties. Whereas, under more favorable climatic conditions (higher radiation and lower temperatures of an early-spring cycle) the FL combined with EC 3.5 could be a trade-off between yield and product quality in terms of appearance, nitrate and nutritional compound contents.

Irrespectively of climate, it is not advisable to apply a NS at 3.5 dS m^−1^ in EF as a decline in productivity is expected, despite a further reduction in nitrate content.

In the more salt-tolerant *C. endivia*, no substantial changes are expected in productivity with the EF, EC3.5 or EF/EC3.5 treatments. Nitrate can be controlled both in endive and escarole in the winter, but this is not true under high growth-promoting climate (high temperature and radiation as in late-spring) when there is an accumulation of nitrates, irrespective of the salt-stress deriving from the cultivation system or NS EC. This response in *C. endivia* deserves further investigation, which should also involve other baby-leaf vegetables grown under Mediterranean greenhouse conditions in late-spring cycles. The deprivation of nutrient solution for a few days before harvest applied under conditions promoting nitrate accumulation (autumn-winter period at EC2.5), may be a feasible strategy to reduce nitrate levels in baby-leaf lettuce and escarole/endive. In floating-grown lettuce, the 3 day-long NS withdrawal has been proved to allow maintain steady production and a concurrent nitrate reduction. However, some concerns arise when it is applied to the EF-plants. In this cultivation system, nitrate accumulation is already inhibited by the salt-stress and its further reduction, implies a detrimental effect on yield, although it may improve leaf thickness. In *C. endivia* the decrease in nitrate content can be achieved with 6 day-long NS withdrawal with an enhanced appearance and antioxidative quality, nevertheless it seems too prolonged as it may provoke a yield reduction. Overall this evidence raise the need for more insights to fine-tune the duration of the NS removal, taking into account the soilless system used and the species-specific characteristics to be both effective in reducing nitrate levels in the product as well as having no or scarce effect on yield.

## Data Availability Statement

The datasets generated for this study are available on request to the corresponding author.

## Author Contributions

GC: conception of the work, data analysis and interpretation, drafting the article, critical revision of the article, and final approval of the version to be published. AB: conception of the work and data collection and analysis. CL: experiments conduction, data collection, and analysis execution. PL: data collection and analysis execution. AE: conception of the work, critical revision of the article, and final approval of the version to be published. All authors contributed to the article and approved the submitted version.

## Conflict of Interest

The authors declare that the research was conducted in the absence of any commercial or financial relationships that could be construed as a potential conflict of interest.
