# Supplementary material for: Reduction of Nitrate Content in Baby-Leaf Lettuce and Cichorium endivia Through the Soilless Cultivation System, Electrical Conductivity and Management of Nutrient Solution
Source: Front Plant Sci. 2021 Apr 29;12:645671. doi: 10.3389/fpls.2021.645671 (PMC8117335; doi:10.3389/fpls.2021.645671)
Supplement: Supplementary file 1 [file Table_1.DOCX]

Table S1- Electrical conductivity (EC) and dissolved O_2_ (DO) measured in the nutrient solution (NS). The initial value of EC and the averaged values of EC and DO, over the whole crop cycle, are reported for each experiment.

| Species | Botanical  variety | Common name | Crop cycle | Soilless cultivation system | Electrical conductivity  of NS  (dS m^-1^) | | | Dissolved O_2_ in the NS  (g L^-1^) |
| --- | --- | --- | --- | --- | --- | --- | --- | --- |
|  |  |  |  |  | Fresh NS | Average of the readings over the whole crop cycle | | |
| *Lactuca sativa* | *longifolia* | Romaine lettuce | Autumn | Ebb and flow | 2.5 | | 2.8 ± 0.2 | 9.9 ± 1.1 |
|  |  |  |  |  | 3.5 | | 4.5 ± 0.1 | 9.3 ± 0.6 |
|  |  |  |  | Floating | 2.5 | | 2.8 ± 0.1 | 8.0 ± 0.9 |
|  |  |  |  |  | 3.5 | | 4.4 ± 0.2 | 8.1 ± 0.9 |
|  |  |  | Early spring | Ebb and flow | 3.5 | | 4.8 ± 0.2 | 9.8 ± 0.6 |
|  |  |  |  | Floating | 3.5 | | 5.0 ± 0.2 | 9.2 ± 0.7 |
| *Cichorium endivia* | *crispum* | Endive | Winter | Ebb and flow | 2.5 | | 2.8 ± 0.1 | 11.3± 0.3 |
|  |  |  |  |  | 3.5 | | 4.5 ± 0.2 | 9.6 ± 0.9 |
|  | *latifolium* | Escarole |  | Floating | 2.5 | | 3.1± 0.2 | 9.7 ± 0.7 |
|  |  |  |  |  | 3.5 | | 4.6 ± 0.4 | 9.1 ± 0.1 |
|  |  |  | Late-spring | Ebb and flow | 3.5 | | 4.4± 0.6 | 10.7± 0.2 |
|  |  |  |  | Floating | 3.5 | | 5.0 ± 0.6 | 8.5± 0.4 |
