# Supplementary material for: Reduction of Nitrate Content in Baby-Leaf Lettuce and Cichorium endivia Through the Soilless Cultivation System, Electrical Conductivity and Management of Nutrient Solution
Source: Front Plant Sci. 2021 Apr 29;12:645671. doi: 10.3389/fpls.2021.645671 (PMC8117335; doi:10.3389/fpls.2021.645671)
Supplement: Supplementary file 2 [file Table_2.DOC]

Table S2 - Effect of the crop cycle and of the genotype on yield, bio-morpho-physiological traits of romaine lettuce leaves. In brackets the standard error of mean.

| Treatments | | Fresh Yield  (kg m^-2^) | Dry yield  (g m^-2^) | Leaf number (n.) | Leaf height  (cm) | Specific leaf area (g cm^-2^) | Chlorophylls  (µg mg^-1^ FW) | hue |
| --- | --- | --- | --- | --- | --- | --- | --- | --- |
| Crop cycle | Genotype |  |  |  |  |  |  |  |
| Autumn | Lastra | 1.3 b^*^  (0.1) | 64 b  (2) | 4.3 b  (0.1) | 13.1 b  (0.2) | 1.6 c  (0.1) | 0.6 c  (0.1) | 125.5 c  (0.1) |
|  | Green Forest | 2.0 a  (0.1) | 103 a  (3) | 4.1 b  (0.1) | 15.6 a  (0.2) | 1.9 b  (0.1) | 0.9 b  (0.0) | 128.2 b  (0.2) |
| Early-spring | Lastra | 1.5 b  (0.1 | 94 a  (4) | 5.4 a  (0.1) | 13.1 b  (0.2) | 2.7 a  (0.1) | 0.6 c  (0.0) | 130.1 a  (0.2) |
|  | Green Forest | 1.5 b  (0.2) | 100 a  (7) | 4.2 b  (0.1) | 11.4 c  (0.3) | 2.6 a  (0.1) | 1.2 a  (0.1) | 133.4 a  (0.2) |

* a-c Means in columns not sharing the same letters are significantly different according to LSD test (*P =* 0*.*05).
