# Supplementary figures and images for: Reduction of Nitrate Content in Baby-Leaf Lettuce and Cichorium endivia Through the Soilless Cultivation System, Electrical Conductivity and Management of Nutrient Solution
Source: Front Plant Sci. 2021 Apr 29;12:645671. doi: 10.3389/fpls.2021.645671 (PMC8117335; doi:10.3389/fpls.2021.645671)

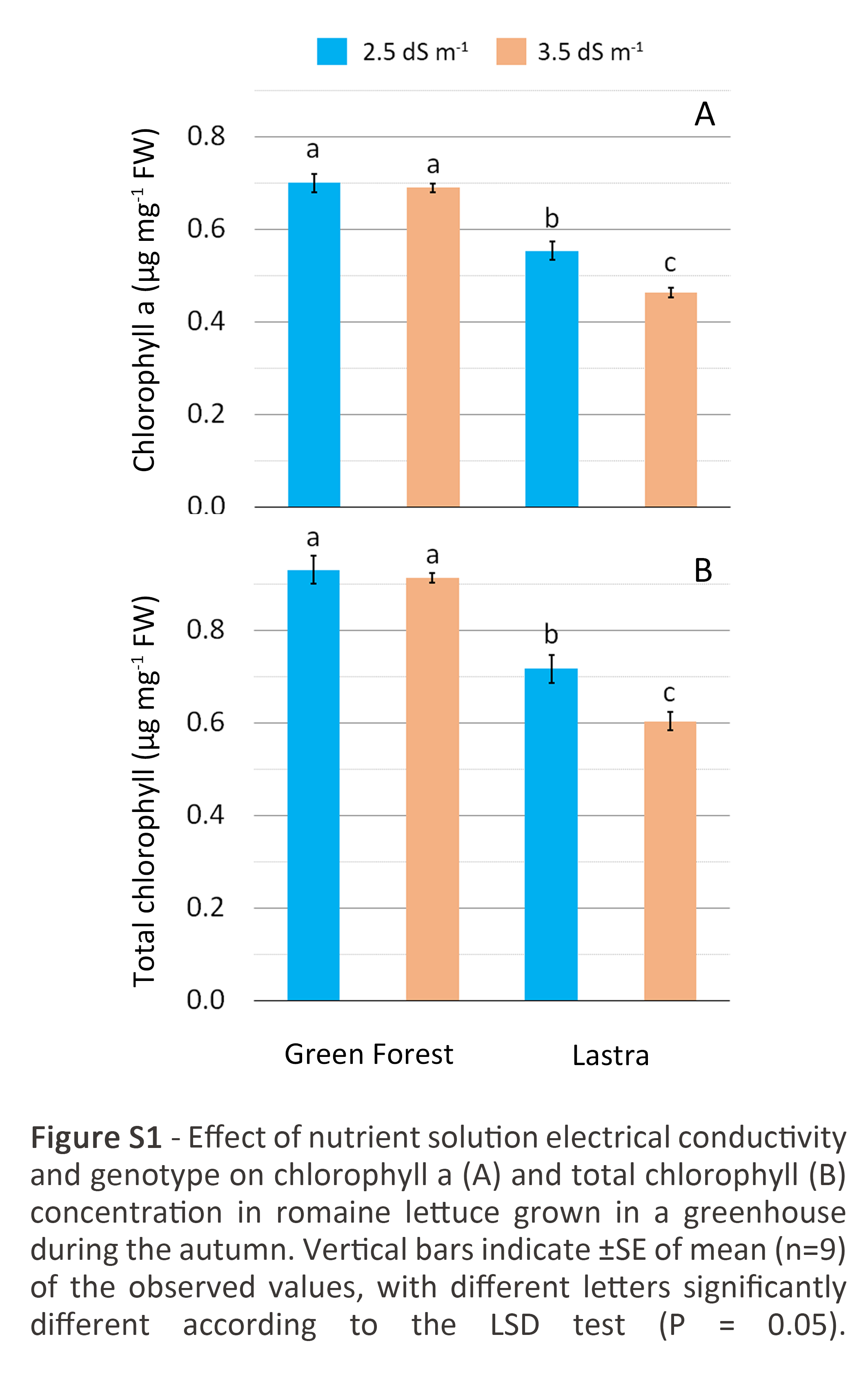

Supplement: Supplementary file 3 [file Image_1.TIF]

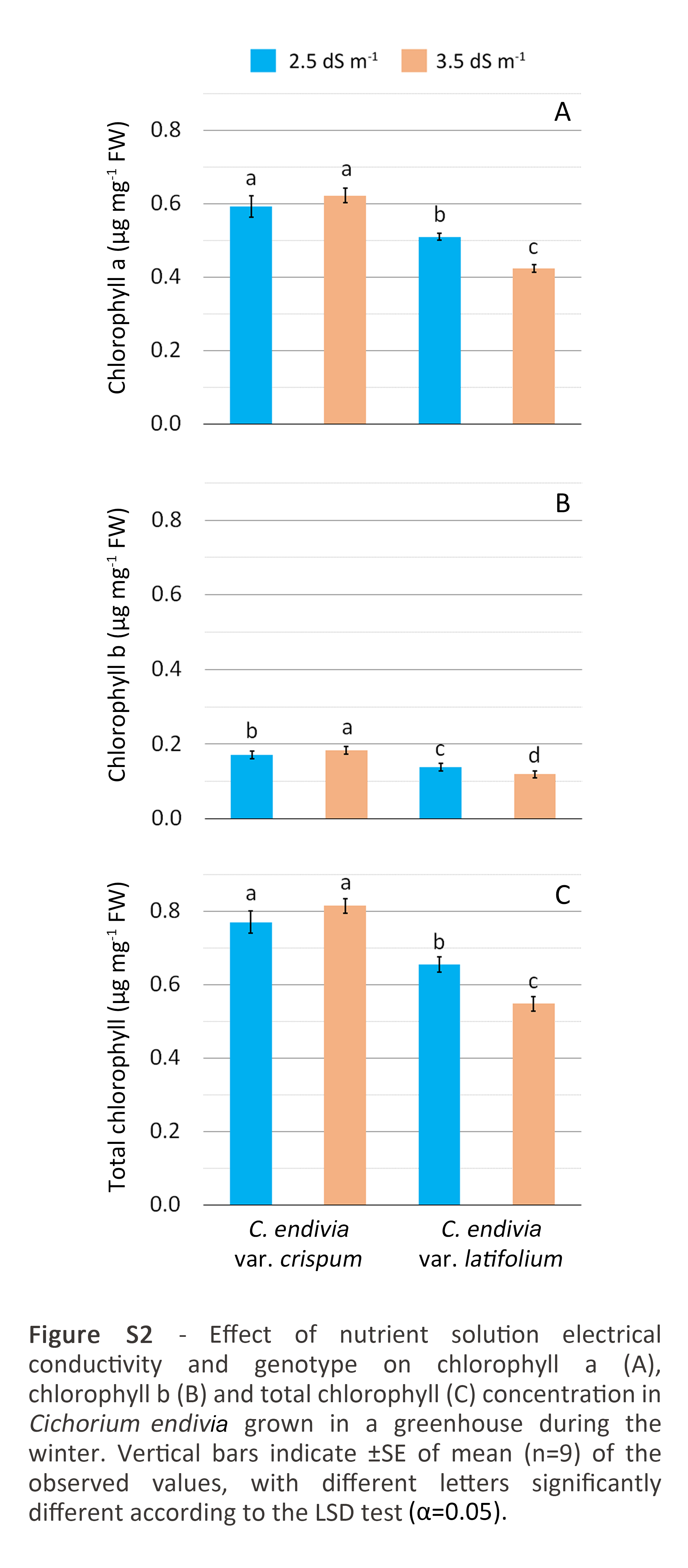

Supplement: Supplementary file 4 [file Image_2.tif]
